# Volatile Phases Derived from Serum, DC, or MLC Culture Supernatants to Deduce a VOC-Based Diagnostic Profiling Strategy for Leukemic Diseases

**DOI:** 10.3390/biom13060989

**Published:** 2023-06-14

**Authors:** Tobias Baudrexler, Tobias Boeselt, Lin Li, Sophia Bohlscheid, Ursel Boas, Christoph Schmid, Andreas Rank, Jörg Schmohl, Rembert Koczulla, Helga Maria Schmetzer

**Affiliations:** 1 Medical Department III, Hospital Großhadern, Ludwig-Maximilians-University, 81377 Munich, Germany; 2 Department of Pulmonary Rehabilitation, German Center for Lung Research (DZL), Phillipps-University of Marburg, 35043 Marburg, Germany; 3 Department of Hematology and Oncology, University Hospital of Augsburg, 86156 Augsburg, Germany; 4 Department of Hematology and Oncology, Diaconia Hospital Stuttgart, 70176 Stuttgart, Germany

**Keywords:** leukemia-derived DC, acute myeloid leukemia, anti-leukemia functionality, leukemia-specific cells, volatile phases above serum and cell culture supernatants, immune monitoring

## Abstract

Volatile organic compounds (VOCs) reflect the metabolism in healthy and pathological conditions, and can be collected easily in a noninvasive manner. They are directly measured using electronical nose (eNose), and may qualify as a systemic tool to monitor biomarkers related to disease. Myeloid leukemic blasts can be transformed into leukemia-derived dendritic cells (DC_leu_) able to improve (anti-leukemic) immune responses. To profile immunological changes in healthy and acute myeloid leukemic (AML) patients’ ex vivo cell cultures, we correlated the cell biological data with the profiles of cell culture supernatant-derived VOCs. DC/DC_leu_ from leukemic or healthy whole blood (WB) were generated without (Control) or with immunomodulatory Kit M (Granulocyte macrophage-colony-stimulating-factor (GM-CSF) + prostaglandin E_1_ (PGE_1_)) in dendritic cell cultures (DC culture). Kit-pretreated/not pretreated WB was used to stimulate T cell-enriched immunoreactive cells in mixed lymphocyte cultures (MLC culture). Leukemia-specific adaptive and innate immune cells were detected with a degranulation assay (Deg) and an intracellular cytokine assay (InCyt). Anti-leukemic cytotoxicity was explored with a cytotoxicity fluorolysis assay (CTX). VOCs collected from serum or DC- and MLC culture supernatants (with vs. without Kit M pretreatment and before vs. after culture) were measured using eNose. Compared to the Control (without treatment), Kit M-pretreated leukemic and healthy WB gave rise to higher frequencies of mature (leukemia-derived) DC subtypes of activated and (memory) T cells after MLC. Moreover, antigen (leukemia)-specific cells of several lines (innate and adaptive immunity cells) were induced, giving rise to blast-lysing cells. The eNose could significantly distinguish between healthy and leukemic patients’ serum, DC and MLC culture supernatant-derived volatile phases and could significantly separate several supernatant (with vs. without Kit M treatment, cultured vs. uncultured)-derived VOCs within subgroups (healthy DC or leukemic DC, or healthy MLC or leukemic MLC supernatants). Interestingly, the eNose could indicate a Kit M- and culture-associated effect. The eNose may be a prospective option for the deduction of a VOC-based profiling strategy using serum or cell culture supernatants and could be a useful diagnostic tool to recognize or qualify AML disease.

## 1. Introduction

### 1.1. Acute Myeloid Leukemia (AML)

AML is a clonal stem cell disorder of the hematopoiesis, which comes with uncontrolled proliferation of myeloid progenitor cells (blasts) [1]. Cytochemistry, immunophenotyping, cytogenetics and molecular biological investigations are usually conducted to confirm diagnosis and to allocate patients to prognostic risk groups [2,3]. The standard treatment of AML leads to high rates of remission; however, there are high rates of relapses in up to 80% of cases in the following two years [4,5,6]. Currently, new treatment strategies based on new chemotherapies or (targeted) passive or active immunotherapies are being developed (e.g., hypomethylating agents, venetoclax) [6,7].

### 1.2. DC-Based Immunotherapy

DCs are professional antigen-presenting cells (APCs). They are activated and maturate after danger-signaling adhesions (e.g., nucleic acids, infectious particles), resulting in upregulated chemokine receptors (e.g., CCR7), MHC-antigens and other costimulatory factors [8,9,10,11]. Ex vivo DCs can be generated from CD14+ monocytes (and loaded with tumor antigens) or from myeloid blasts (DC_leu_, leukemia-derived DC; without induction of blast proliferation) from AML patients’ WB in the presence of different combinations of response modifiers (Kits) [9,12,13]; the resulting DCs express costimulatory molecules together with individual patients’ leukemic antigens, and gain the capacity to activate the cells of the immune system against blasts. Ex vivo-generated (and manipulated) DCs or DC_leu_ could be used for an adoptive transfer; applying Kits directly to AML patients could induce DC_leu_ from blasts, leading to an antileukemic immunoreaction in vivo [9]. Immunomodulatory Kit M, composed of granulocyte macrophage colony-stimulating factor (GM-CSF) and prostaglandin E1 (PGE_1_), has proved to be one of the best combinations of immune response modifiers to generate DC/DC_leu_ from leukemic WB [9,13]; therefore, it was used in this ex vivo study.

### 1.3. Immune System

The key players of the innate immune system are APCs (e.g., monocytes, macrophages, DC), cytokine-induced killer (CIK), invariant natural killer T cells (iNKT cells) and natural killer (NK) cells; they mediate the earliest interactions with pathogens/tumors [12,13,14]. Antigen-specific response and generation of immunologic memory are the central tasks of the adaptive immune system [12,13]. After activation, naive T cells (T_naive_, CD3+CD45RO−) are probably converted to non-naive T cells (T_non-naive_, CD3+CD45RO+), which mediate various immune responses or develop into long-living central memory cells (T_cm_, CD3+CD45RO+CD197+) or effector memory cells (T_em/eff_, CD3+CD45RO+CD197−) to facilitate a faster reactivation of the immune system against recurring antigens [8,15]. These immunoreactive cells can be detected using flow cytometry (abbreviations are given in Table 1).

### 1.4. Leukemia-Specific Cells and Antileukemic Process

The degranulation assay (Deg) allows the detection and quantification of lysosomal-associated membrane glycoproteins (LAMPs), such as LAMP-1 (CD107a), which are involved in granzyme/perforin-associated degranulation granules. The intracellular cytokine assay (InCyt) allows the intracellular (antigen specific) quantification of cytokines (interferon gamma (INFy) and tumor necrosis factor alpha (TNFa) on a single-cell level, representing triggers of immune responses and mediators of cell apoptosis [12,15,21]. Cytotoxicity assays (CTX) evaluate the antileukemic potential of stimulated and unstimulated effector cells [12]. The Deg assay in combination with the InCyt assay and the CTX provides a refined and complex analysis of the functionality of cells, especially with respect to immune cells’ (leukemia-specific) activity and cytotoxicity [12].

### 1.5. Methodological Tools to Monitor AML Disease or Antileukemically Related Processes

Analyses of different (activating or inhibitory) cellular/humoral, soluble factors, or even the smallest molecules could contribute to our understanding of leukemia-related as well as antileukemic processes [8,15,21,22,23,24]. In recent years, the role of physical factors (e.g., physiological hypoxia [25] or circulating vesicles (e.g., extracellular vesicles (EVs) [26,27]) has been tested with respect to a refined monitoring of immunological or tumor-associated processes.

### 1.6. VOC Analysis as a New Option to Characterize and Monitor (Malignant) Diseases

Every creature (human, plants, animals) emits organic compounds into the environment. Since exhaled molecules reflect the metabolism in healthy and pathological conditions, exhaled air may qualify as a systemic tool to monitor biomarkers related to disease [28]. VOCs are organic chemicals containing hydrocarbon compounds. Exhaled volatile organic compounds (VOC) can be collected easily, in a noninvasive matter (either by collecting exhaled breath directly into an electronical nose (eNose) or by analyzing collected VOCs bound to carriers (fleeze or earloop masks)), and afterwards analyzed using an eNose. Proof that VOC analyses can contribute to detecting disease-associated endogenic metabolic or cell-derived molecular VOC markers, e.g., in Parkinson’s, Alzheimer’s, lung cancer or AML, has been presented [29,30,31,32]. 

Although applications of VOC-detecting technologies are still diverse, promising results in the profiling of breath-derived VOC patterns have contributed to differentiating breath from patients with vs. without lung cancer, and from (COPD) patients with vs. without an alpha-1 deficiency. It is even possible to detect COVID-19 infection vs. no infection using VOC analyses of patients’ urine samples [31,33,34,35], and to identify patients with vs. without major depression [36]. Moreover, specific VOC analyses have been shown to be a promising tool for detecting bladder tumors, using measurements of urine-derived VOCs [37]. Moreover, VOCs collected above cell culture supernatants have been shown to correlate with subtypes of the underlying disease [38].

The aims of this trial were as follows:

1. Generation and quantification of DC/DC_leu_ (subpopulations) using Kit M-treated (vs. untreated) WB from AML patients and healthy volunteers;
2. Characterization of (activated) immune cells before (uncultured MLC) and after MLC (with Kit M-pretreated vs. untreated WB);
3. Detection and quantification of antileukemic/leukemia-specific innate and adaptive immune cells using Deg and InCyt assays, or CTX after MLC;
4. VOC analyses above collected serum and cell culture supernatants (DC, MLC, with/without Kit M treatment) using eNose;
5. Correlation of cell biology with the VOC results, and potentially the deduction of a VOC-based profiling strategy using serum or cell culture supernatants

## 2. Material and Methods

### 2.1. Cell Biological Experiments

#### 2.1.1. Sample Collection

The sample acquisition for this work was conducted between 2019 and 2021 through the University Hospitals of Munich, Oldenburg, Augsburg, the Rotkreuzklinikum in Munich, and the Diakonieklinikum in Stuttgart. After patients’ and donors’ written agreement to experimental use of their blood donation, WB samples were collected in syringes containing standardized concentrations of heparin (7.5 mL, Sarstedt, Nuembrecht, Germany) from patients in acute phases of AML, and from healthy volunteers. This was in consensus with the Declaration of Helsinki and the local ethics committee of LMU in Munich (Pettenkoferstr. 8a, 80336 München, Ludwig-Maximilian University Hospital, Munich; vote no. 339-05). The patients’ diagnostics of clinical findings were provided by the cooperating hospitals.

#### 2.1.2. Patients’ Characterization

WB samples were collected from AML patients (*n* = 17) and healthy volunteers (*n* = 14). On average, AML patients were 61 (range 29–98), and healthy volunteers were 30 years old (range 22–58). The female-to-male ratio in AML patients was 1:0.55, and in healthy volunteers was 1:1. AML patients’ samples were characterized using the French American British (FAB) classification (as far as possible and available), and assigned to primary (pAML) or secondary AML (sAML). AML patients were sub-grouped into stages of the disease (first diagnosis, persisting disease, relapse after stem cell transplantation (SCT)) and risk groups (EuropeanLeukemiaNet (ELN) risk stratification) [38]. Moreover, blast phenotypes and blood parameters (white blood cells, platelets, hemoglobin in PB) were collected on the day of sampling. Nine patients presented at first diagnosis, five patients with persisting disease and three patients with relapse after SCT. An overview is presented in Table 2.

The cellular composition of blood samples from AML patients was 32.18% blasts (range 11–79), 14.38% T cells (range 1.85–56.00), 3.25% NK cells (range 0.9–6.3), and 1.3% CIK cells (range 0.56–4.60). The cellular composition of blood samples from healthy volunteers was 8.49% monocytes (range 4.55–14.64), 16.91% T cells (range 10.45–44.80), 6.5% NK cells (range 4.34–9.30), and 1.5% CIK cells (range 0.32–3.21). In cases of aberrant expression of lineage markers on blasts, these markers were not included.

#### 2.1.3. Cell Characterization by Flow Cytometry

To evaluate and quantify phenotypes of DC/DC_leu_, leukemic blasts, monocytes and immune reactive cell subsets of the adaptive and innate immunity analyses were conducted via flow cytometry, using a fluorescence-activating cell-sorting flow cytometer (FACSCalibur^TM^). Using a refined gating technique and the analysis software CellQuestPro (Becton Dickinson, Heidelberg, Germany), the functionalities of cells (proliferation, cytokine production, degranulation and cytotoxicity) could be investigated [12,15]. Panels with various monoclonal antibodies (moAbs) labelled with fluorescein isothiocyanate (FITC), phycoerythrin (PE), phycoerythrin cyanin 7 (PCy7) or allophycocyanin (APC) were used, provided by Becton Dickinson, Heidelberg, Germany^a^, Beckman Coulter, Krefeld, Germany^b^, Santa Cruz Biotechnology, Heidelberg, Germany^c^, and Bio Legend, San Diego, CA, USA^d^. Clone numbers are given in brackets. For the detection of CD3^a^ (HIT3a 55339), CD4^a^ (SK3 345768 BD), CD14^b^ (RMO52 B36297), CD15^b^ (80H5 B36298), CD19^b^ (REA675 5200608927), CD34^b^ (581 IM1870), CD45RO^b^ (UCHL1 IM1247U), CD71^b^ (YDJ1.2.2 IM0483), CD117^d^ (104D2 313232), CD197^d^ (REA546 130-099-174), CD107a^d^ (H4A3 328606), and IPO38^c^ (E2108), FITC moAbs were used. PE-conjugated moAbs were used for the discovery of CD3^b^ (UCHT1 A07747), CD4^a^ (RPA-T4 555347), CD80^b^ (MAB104 IM1976U), CD206^b^ (3.29B1.10 IM2741), and INFy^d^ (RUO XMG1.2). PCy7-labelled moAbs against CD3^b^ (UCHT1 737657), CD4^b^ (SFCI12T4D11 737660), CD14^a^ (M5E2 557742), CD20^b^ (B9E9 IM3629), CD34^b^ (581 A21691), CD56^b^ (N901 A21692), CD117^b^ (104D2D1 B49221), CD197^a^ (3D12 557648 BD), and TNFa^d^ (Mab11 502930), and moAbs labelled with APC against CD3^b^ (UCHT1 IM2467), 6B11^d^ (6B11 342908), CD14^b^ (RMO52 IM2580), CD15^a^ (HI98 551376), CD19^b^ (J3-119 IM2470), CD20^b^ (B9E9 A21693), CD34^b^ (581 IM2472), CD45RO^d^ (UCHL1 304210), CD56^b^ (N901 IM2474), CD69^a^ (FN50 555533), CD80^d^ (2D10 305220), CD117^b^ (104D2D1 B36300), and CD206^a^ (19.2 550889 BD) were utilized for detection. Isotype controls were included according to the manufacturer’s instructions [39]. 7AAD^a^ (RUO 559925 BD) was used to distinguish between non-viable and viable cells.

#### 2.1.4. Staining and Measurement

Before or after culture, cells were stained using fluorochrome-labeled monoclonal antibodies, and were quantified as described [12,21]. Additionally, for intracellular staining (e.g., IPO38, INFy, TNFa) cell fixation and cell permeabilization were performed with Medium A (FIX&PERM^®^, Thermo Fisher Scientific, Waltham, MA, USA) and Medium B (FIX&PERM^®^, Thermo Fisher Scientific, Waltham, MA, USA). Generated DC/DC_leu_ were stained with patient-specific blast markers (e.g., CD15, CD34, CD56, CD65, CD117), and with antibodies against DC typical markers (e.g., CD80, CD206), using DC markers not expressed on ‘uncultured’ blasts [12,15]. The expression of CD197 (CCR7) determined mature DCs (DC_mat_). Moreover, proliferating blasts and monocytes were defined by the co-expression of blast markers (or monocyte-specific surface markers in healthy samples), together with CD71 or IPO38 (proliferation markers) without co-expression of DC markers [8]. Abbreviations are given in Table 1.

#### 2.1.5. Preparation of Cells

AML or healthy WB was either directly used for experiments (the workup of all blood samples was routinely carried out under a hood), and mononuclear cells (MNC) and T cells were isolated and frozen for later use. The MNCs isolated from the WB (according to standard preparations [12]) were used for the isolation of T cells via MACS microbead technology, based on a CD3 magnetic cell selection (CD3 Microbeads, Milteney Biotech, Bergisch Gladbach, Germany), as described in the manufacturer’s instructions [40]. T cell purity, verified by flow cytometry, was on average 91% ± 8.2% in healthy WB and 84% ± 9.3% in AML samples. Isolated T cells were further quantified with trypan blue (Biochrom, Berlin, Germany), counted (Neubauer counting chambers), and resuspended in 1 mL RPMI/PS (Penicillin/Streptomycin) (for use with T cells in MLC) or in 1 mL cytotoxicity medium containing 85% RPMI/PS and 15% human serum (CTX medium, for use as a target MNC in the CTX assay) [8,12].

#### 2.1.6. Dendritic Cell Culture (DC Culture)

DC and DC_leu_ were cultured as described [8]. For stimulation of the blasts’ differentiation into DC_leu_, response modifiers (‘Kit M’) were added before and during culture, as a restimulation, on day 2/3 [12]: Kit M contained 800 U/mL granulocyte macrophage colony-stimulating factor (GM-CSF, Sanofi-Aventis, Frankfurt, Germany), and 1 μg/mL prostaglandin E1 (PGE1, Santa Cruz Biotechnology, Dallas, TX, USA) [22]. WB not treated with response modifiers served as a Control. WB cultures were incubated for 7–8 days under physiological conditions (37 °C, 5% CO_2_, 21% O_2_ and 95% humidity) [12].

#### 2.1.7. Mixed Lymphocyte Culture (MLC Culture)

DC cultures were harvested on day 7–8, and MLC cultures were set up as described [12]. For stimulating the immune reactive cells, 5 μL of 50 U/mL interleukin 2 (IL-2, PeproTech, Berlin, Germany) was added on day 0 of MLC cultures. On day 2/3 and 5/6, restimulations with IL-2 were conducted for every single well. Cell analyses before culture served as the Control (‘uncultured MLC’). Under physiological conditions (37 °C, 5% CO_2_, 21% O_2_ and 95% humidity), the MLC was incubated for 7 days. After culture, measurements were carried out with Kit M (MLC^WB-DC(Kit-M)^) and Control (MLC^WB-DC(Control)^), and cells were used for the Deg-, the InCyt- and the CTX-assays [8]. Different immune cell subtypes after MLC were quantified using flow cytometry. Abbreviations are given in Table 1.

#### 2.1.8. Degranulation Assay (Deg) and Intracellular Assay (InCyt)

Deg and InCyt cultures were set up as described [12] to detect leukemia-specific cells, as given in Table 1. AML samples were stimulated (or not stimulated) in parallel with two leukemia-associated antigens (LAA): 2 μg/mL Wilms tumor 1 protein (WT-1, PepTivator^®^, Miltenyi Botech, Bergisch Gladbach, Germany) and 2 μg/mL PRAME (‘Melanoma antigen preferentially expressed in tumors’, UniProt ID: P78395, PepTivator^®^, Miltenyi biotech, Bergisch Gladbach, Germany). Healthy cells were stimulated/not stimulated with 10 μg/mL staphylococcal enterotoxin B (SEB, Sigma-Aldrich, St. Louis, MO, USA). Cultures without antigen stimulation served as a negative Control (‘Unstimulated’) [12].

#### 2.1.9. Degranulation Assay (Deg)

A FITC-conjugated antibody against CD107a (Bio Legend, San Diego, CA, USA) was used to detect cell degranulation as a marker of cell cytotoxicity [20]. After one hour of incubation, 2 μg/mL Monensin solution (Bio Legend, San Diego, CA, USA) was added to the culture according to the manufacturer’s instructions to avoid loss or weakening of FITC-CD107a antibodies’ fluorescence due to endosomal or lysosomal reinternalization [41]. Afterwards, the culture was incubated under physiological conditions (37 °C, 5% CO_2_, 21% O_2_ and 95% humidity) for an additional 15 h. After incubation, cells were harvested, centrifuged, resuspended in PBS/FCS (phosphate-buffered saline/fetal calf serum), and stained with antibodies [12,15]. 

#### 2.1.10. Intracellular Assay (InCyt)

Production of tumor necrosis factor alpha (TNFa) [42] and interferon gamma (INFy) [12] was used to analyze intracellular cytokine production. The cells were stained with PCy7-conjugated TNFa antibodies (Bio Legend, San Diego, CA, USA) and PE-conjugated INFy antibodies (Bio Legend, San Diego, CA, USA). Cellular cytokine production was stopped after one hour of incubation with 5 μg/mL Brefeldin A solution (Bio Legend, San Diego, CA, USA), according to the manufacturer’s instructions. Afterwards, the culture was incubated for 15 h, and cells were harvested, centrifuged, resuspended in PBS/FCS, and stained with antibodies [12,15].

#### 2.1.11. Cytotoxicity Fluorolysis Assay (CTX)

To investigate the ability of effector cells (T cell-enriched cells stimulated with or without Kit M- treated WB after MLC) to lyse target cells (thawed viable patients’ MNCs stained with two different blast markers), a cytotoxicity fluorolysis assay was conducted as described [12]. For each test, equal amounts of effector cells and target cells were mixed. Afterwards, these tubes were incubated for 0, 3 and 24 h in standard physiological conditions (37 °C, 5% CO_2_, 21% O_2_ and 95% humidity). For the control group, effector and target cells were separately incubated and mixed shortly before measurement. By adding 7AAD (Becton Dickinson, Heidelberg, Germany) and fluorosphere beads (Beckman Coulter, Krefeld, Germany), the cytotoxic activity of effector cells could be evaluated through quantification of viable target cells. The lytic activity of effector cells was calculated and defined as the percentage of viable target cells in the culture with co-cultured effector and target cells (for 3 h and 24 h), as compared to Control [8]. 

**Scheme 1:**
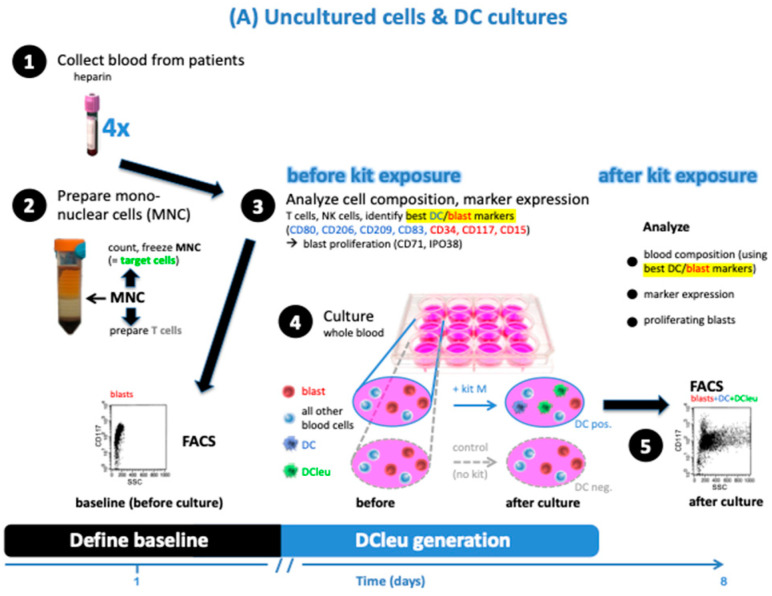

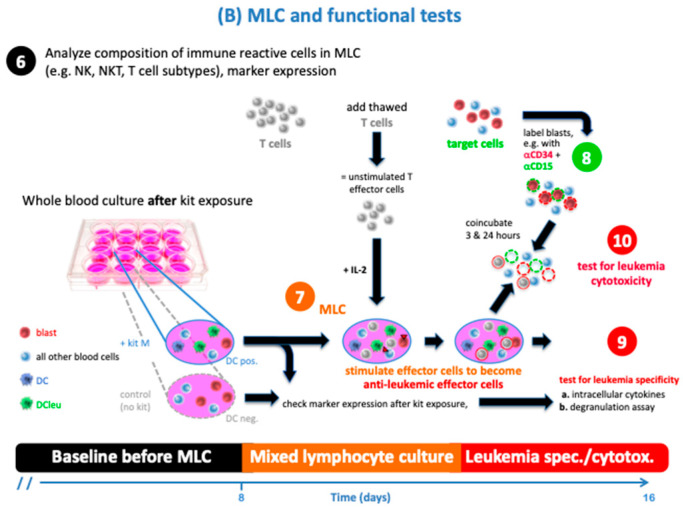
Set up of cell biological experiments. 1. Heparinized blood samples from healthy and leukemia probands were used for cell biological experiments; 2. MNC (mononuclear cells) and T cells were gained from WB and later used for MLC (‘T cell-enriched’ mixed lymphocyte culture) and CTX; 3./4./5. DC cultures were set up with leukemic and healthy WB and measured using flowcytometry before and after culture with (vs. without) Kit M; 6./7./8. MLC cultures were set up with harvested DC culture + T cells, stimulated with IL-2, and measured using flowcytometry before and after culture; 9./10. Several immune assays were performed after MLC: DEG + InCyt, as well as a CTX assay after the mixture of MNC and MLC.

An overview of the cell biological experiments is given in the following Scheme 1.

### 2.2. VOC Experiments

#### 2.2.1. Collection of Serum and Cell Culture Supernatant

As a source for VOC analyses, we collected serum as well as cell culture supernatants from healthy and AML sample donors. Cell culture supernatants were taken from DC cultures and MLC cultures (see above); these were the supernatants of DC cultures before culture with and without the addition of Kit M (DCD0 M/DCD0 ø), or after culture (DCDE M/DCDE ø), and the supernatants of MLC cultures before culture with or without WB pretreated with Kit M (MLCD0 M/MLCD0 ø), or after culture (MLCDE M/MLCDE ø). An overview of the collected supernatants of each proband is given in Table 2. For the preparation of cell culture supernatants, 1 mL of DC/MLC cell suspension was centrifuged at 480× *g* and 4 °C for 5 min, and the supernatants were again centrifuged at 2000× *g* and 4 °C for 10 min. Afterwards, supernatants were collected and stored in 300–500 μL aliquots in 1.5 mL safe-lock tubes (Eppendorf Tubes^®^, Hamburg, Germany), frozen, and stored at −80 °C.

For preparation of serum supernatants, 4 mL serum was collected (S-Monovette^®^, Sarstedt, Nuembrecht, Germany) and centrifuged at 1200× *g* for 10 min at 4 °C; afterwards, supernatants were centrifuged again at 1800× *g* for 10 min at 4 °C. Aliquots of 300–500 μL were filled in 1.5 mL safe-lock tubes (Eppendorf Tubes^®^, Hamburg, Germany), frozen, and stored at −80 °C [43].

Collected serum and culture supernatants were sent on dry ice to the analytical laboratory of the Marburg University Hospital, where the VOC analyses were conducted.

#### 2.2.2. Experimental Set-Up of the Cyranose 320

**Scheme 2:**
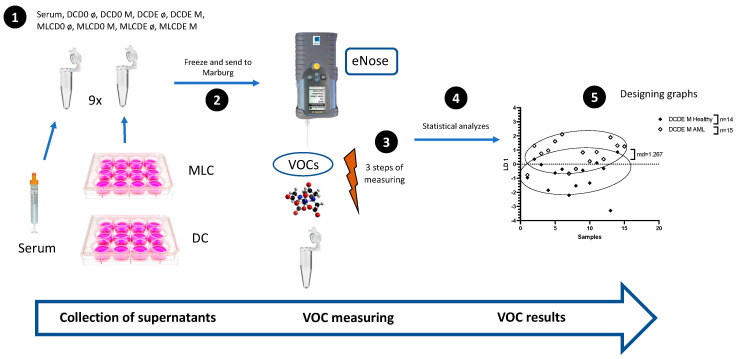
Overview of the VOC experiments. 1. Collection of supernatants of serum, DC- and MLC cultures; 2. Freezing, transportation to Marburg and thawing of the tubes; 3. Measurement of VOCs above supernatants using the eNose; 4./5. Statistical analyses of VOC data and presentation of VOC differentiations in graphs.

Serum and cell culture supernatants were thawed in Marburg, and afterwards, VOCs were collected from the surface above every single supernatant (healthy, leukemia, serum, DC- or MLC-samples) and measured in a sealed room using the Cyranose 320 electronic nose (eNose) [43,44]. The Cyranose 320^®^ belongs to the group of polymers sensors and consists of 32 thin-film carbon polymer chemiresistors (NoseChip). The sensor response is based on binding volatile organic components (VOCs) depending on structure, size, polarity and proton affinity. The sensors convert chemical signals to electronical signals [45]. Exposure to gases causes the polymer layer to swell while the analyte is absorbed. Each sensor responds differently to an analyte; conversely, no sensor usually responds to only a single analyte. The magnitude of a sensor’s response depends on the doping of the sensor and the physicochemical character of the analyte. For single substances or for a mixture, respectively, characteristic pattern smellprints are produced; these are composed of 32 individual signals. The measurement is based on the change in the resistance of each sensor when exposed to volatile organic gases. In this process, the medical air did not have any effect on the VOCs to be measured. In this context, it rather stood for a standard to not allow any contamination by the ambient air. To ensure that the sensors were working correctly in each study, defined olfactory substances were used to check their functions. Only when all 32 sensors of the eNose indicated correct reference ranges could the study begin. Before each measurement series, the sensors were trained with the so-called training set. The sensors were calibrated once a month with a test battery of odors. Every single measurement was performed in three steps. A reference value was determined using medicinal air (Aer medicinalis, Linde Gas Therapeutics GmbH, Unterschleißheim, Germany) (1). After 60 s of step 1, the VOCs on the volatile surface of the cell supernatants were measured (2), followed by a cleaning step of the sensors of the eNose with exposed ambient air within another 60 s (3) [31]. All samples were measured in triplicate, and arithmetic mean values were used for principal component analysis. Afterwards, linear discriminant analyses (LD) were performed, and the LD results were used for further analyses, particularly contingency table analyses. The Mahalanobis distance between groups was then determined [43,44]. An overview of the VOC sampling, measurement and analysis is given in the following Scheme 2. 

### 2.3. Statistical Methods

All cell biological measurements were conducted using flow cytometry (FACSCalibur^TM^, Becton Dickinson, Heidelberg, Germany) and the software BD CellQuestPro (Becton Dickinson, Heidelberg, Germany). Statistical analyses were conducted with Excel (Microsoft^®^ Excel, version 16.52, Redmond, WA, USA) and Prism 9 (GraphPad Software, version 9.1.1, San Diego, CA, USA). Data are presented as mean ± standard deviation. Statistical comparisons of two groups were performed using multiple two-tailed *t*-tests.

Statistical investigations of VOC data calculated via linear discriminant analyses were conducted with Prism 9 (GraphPad Software, version 9.1.1, San Diego, CA, USA). Data are presented as sensitivity, specificity, a positive predictive value (PPV) and a negative predictive value (NPV). Statistical comparisons for contingency table analyses were conducted with Fisher’s exact test. Moreover, differences were considered highly significant with *p* values ≤ 0.005, significant with *p* values ≤ 0.05, and borderline significant with *p* values 0.05 to 0.10.

## 3. Results

### 3.1. Cell Biological Results

DC/DC_leu_ were generated by treating blasts (monocytes) in leukemic and healthy WB with (vs. without) blast-modulating Kit M. DCs and their subtypes (DC_leu_, DC_mat_, DC_leu-mat_) were quantified after culture with (DC^WB(Kit M)^) or without Kit M (DC^WB(Control)^). Afterwards, DC/DC_leu_-containing samples were used to stimulate T cell-enriched immune cells in MLC. The composition of the immune reactive cells was analyzed before (uncultured MLC) and after MLC (MLC^WB-DC(Control)^ or MLC^WB-DC(Kit M)^). Additionally, specific antileukemic effects were studied via Deg and InCyt. The cytotoxic impact of T cell-enriched MLC (with and without pretreatment with Kit M) was evaluated via CTX in AML samples. Abbreviations for cell populations are given in Table 1. An overview of the experiments is given in Scheme 1.

 

**Successful generation of DC/DC_leu_ from healthy and AML WB**

**Significantly higher frequencies of (mature) DC and their subtypes were generated with (vs. without) Kit M-pretreated healthy WB compared to Control**

**Figure 1:**
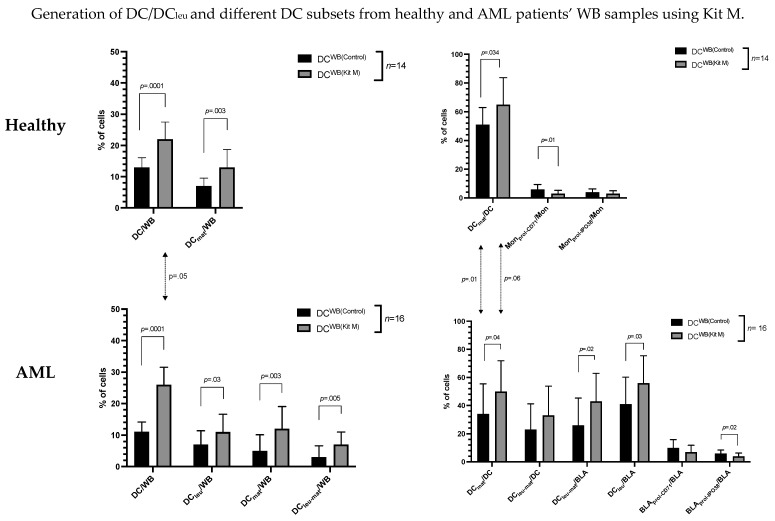
DC/DC_leu_ generation from AML and healthy WB: ‘**left side**’ shows the average frequencies ± standard deviation of generated DC (subtypes) in healthy and leukemic WB fractions (with and without Kit M-pretreated WB (DC^WB(Control)^; DC^WB(Kit M)^)). ‘**right side**’ shows the average frequencies ± standard deviation of generated DC subtypes, proliferating blasts and monocytes for AML or healthy samples in different cell subtype fractions (with and without Kit M-pretreated WB (DC^WB(Control)^; DC^WB(Kit M)^)). Statistical tests were performed using multiple *t*-tests. Differences were considered as highly significant with *p* values ≤ 0.005, significant with *p* values ≤ 0.05, and borderline significant with *p* values between 0.05 and 0.10. Abbreviations of cell types are given in Table 1.

Using healthy samples, we could generate significantly higher frequencies of DC and DC_mat_ within WB when using DC^WB(Kit M)^ rather than DC^WB(Control)^ (%DC/WB: Kit M: 22 ± 5.47; Control: 13 ± 3; *p* = 0.0001, and %DC_mat_/WB: Kit M: 13 ± 5.73; Control: 7 ± 3; *p* = 0.003 or %DC_mat_/DC: Control: 28 ± 10; Kit M: 65 ± 19; *p* = 0.034). Proliferation of monocytes (as detected by co-expression of CD71 or IPO-38) from healthy WB was not induced under the influence of Kit M (Figure 1 ‘healthy’). 

In leukemic samples, we found significantly higher frequencies of mature and leukemia-derived DC (subsets) in DC^WB(Kit M)^, compared to DC^WB(Control)^ (e.g., %DC/WB: Kit M: 26± 5.55; Control: 16 ± 3; *p* = 0.0001, and %DC_leu_/WB: Kit M: 11 ± 5.58; Control: 7 ± 4; *p* = 0.03% or DC_mat_/WB: Kit M: 12 ± 7.06; Control: 5 ± 5; *p* = 0.003). We found significantly increased frequencies of DC subsets in DC^WB(Kit M)^ compared to DC^WB(Control)^ (e.g., %DC_mat_/DC: Kit M: 50 ± 22; Control: 34 ± 21; *p* = 0.04, and %DC_leu-mat_/BLA: Kit M: 43 ± 20; Control: 26 ± 19; *p* = 0.02 and %DC_leu_/BLA: Kit M: 56 ± 19; Control: 41 ± 19; *p* = 0.03). Proliferation of blasts (as detected in the co-expression of CD71 or IPO-38) from leukemic WB was not induced by Kit M treatment (Figure 1 ‘AML’).

 

**Significantly higher frequencies of DC (subtypes) were found in healthy vs. AML WB samples after Kit M treatment**

We found significantly higher frequencies of DC (subtypes) in leukemic compared to healthy WB samples under the influence of Kit M (e.g., %DC/WB: healthy: 22 ± 5; leukemia: 26 ± 6; *p* = 0.05). Significantly higher frequencies of DC_mat_/DC were found in healthy (vs. leukemic) DC^WB(Control)^ (%DC_mat_/DC: healthy: 51 ± 12; leukemia: 34 ± 21; *p* = 0.01).

In summary, we found higher frequencies of DCs and their subtypes in Kit-treated healthy and leukemic WB when compared to Control, while we could not detect an induction of blast or monocyte proliferation after Kit M treatment. Moreover, we conclude that Kit M-pretreated healthy WB gave rise to higher frequencies of DCmat compared to Kit M-pretreated AML samples.

 

**Stimulation of immune cells was successful after healthy and leukemic MLC culture compared to uncultured MLC**

**Figure 2:**
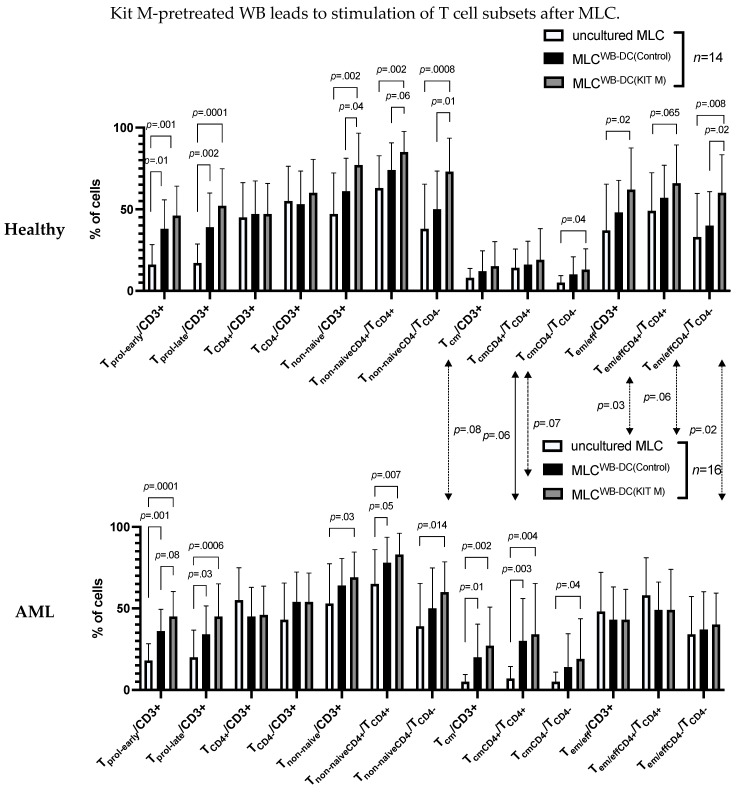
Stimulatory effect of DC/DC_leu_ generated with (vs. without) Kit M from healthy or AML WB on the composition of immunoreactive cells in mixed lymphocyte culture (MLC). The average frequencies ± standard deviation of T cell subsets after stimulation of T cell-enriched immunoreactive cells containing Kit M-pretreated WB (MLC^WB-DC(Kit M)^) from leukemic or healthy WB compared to WB not pretreated with Kit M (MLC^WB-DC(Control)^) and uncultured cells (uncultured MLC) are given. Statistical tests were performed using multiple *t*-tests. Differences were considered highly significant with *p* values ≤ 0.005, significant with *p* values ≤ 0.05, and borderline significant with *p* values between 0.05 and 0.10. Abbreviations of cell types are given in Table 1.

After MLC culture (vs. uncultured MLC) with healthy or leukemic samples, we found significantly higher frequencies of proliferating, activated T cells (T_prol-early_/CD3+, T_prol-late_/CD3+, T_non-naive_/CD3+, T_non-naiveCD4+_/T_CD4+_, T_non-naiveCD4−_/T_CD4−_), and memory T cells (T_em/eff_/CD3+, T_em/effCD4+_/T_CD4+_, T_em/effCD4−_/T_CD4−_) (T_cm_/CD3+, T_cmCD4+_/T_CD4+_, T_cmCD4−_/T_CD4−_) (Figure 2).

 

**Significantly higher frequencies of T cell subsets were found in MLC^WB-DC(Kit M)^ compared to MLC^WB-DC(Control)^ in healthy and AML samples**

In healthy samples, we found significantly higher frequencies of non-naïve T cell subsets and of T_em/effCD4−_/T_CD4−_ in MLC^WB-DC(Kit M)^ compared to MLC^WB-DC(Control)^ (%T_non-naïve_/CD3+: Kit M: 77 ± 20; Control: 61 ± 20; *p* = 0.04 and %T_non-naiveCD4−_/T_CD4−_: Kit M: 73 ± 21; Control: 50 ± 23; *p* = 0.01 and %T_em/effCD4−_/T_CD4−_: Kit M: 60 ± 23; Control: 40 ± 21; *p* = 0.02). In leukemic samples, we found borderline significantly higher frequencies of T_prol-early_/CD3+ in MLC^WB-DC(Kit M)^ compared to MLC^WB-DC(Control)^ (%T_prol-early_/CD3+: Kit M: 45 ± 15; Control: 36 ± 13; *p* = 0.08), and (non-significantly) increased frequencies of T_cm_/CD3+ (Figure 2).

 

**Significant differences were found between healthy and AML patients’ T cell subsets**

In healthy samples (compared to AML patients’ samples), we found significantly higher frequencies of T_em/eff_/CD3+ and T_em/effCD4−_/T_CD4−_ after MLC^WB-DC(Kit M)^ (%T_em/eff_/CD3+: healthy: 62 ± 26; leukemia: 43 ± 19; *p* = 0.03 and %T_em/effCD4−_/T_CD4−_: healthy: 60 ± 23; leukemia: 40 ± 19; *p* = 0.02) (Figure 2).

We summarize that IL-2 had a significant effect on the provision, proliferation and activation of T cells after culture compared to uncultured T cells. Moreover, Kit M-treated WB had a (significant) impact on the proliferation and activation of healthy and AML T cells. Additionally, we found significantly higher frequencies of memory T cell subsets in healthy samples compared to AML patients’ samples.

 

**Degranulation and intracellular assay results**

**Figure 3:**
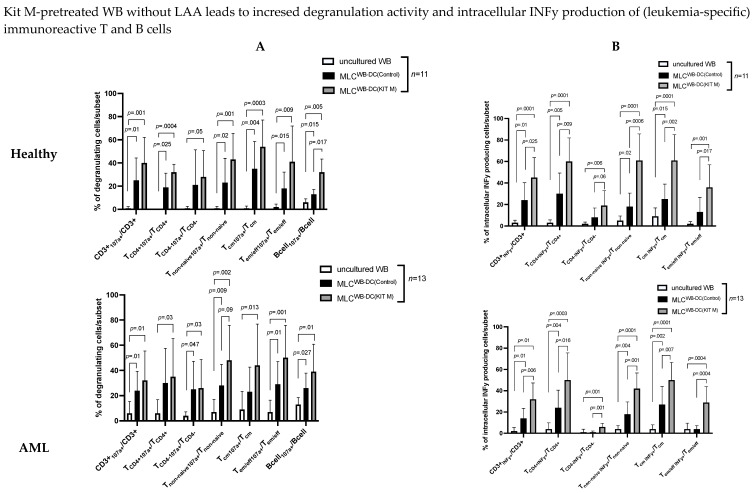
Stimulatory effect of Kit M-pretreated WB on (leukemia-specific) immunoreactive T cell subsets and B cells. Presented herein are the degranulation activities (**A**) and intracellular INFy production of adaptive immune cells (**B**) of leukemic or healthy WB (uncultured WB), before and after culture of leukemic and healthy MLC pretreated with (MLC^WB-DC(KIT M)^) or without Kit M (MLC^WB-DC(Control)^), without LAA stimulation. Also given are the average frequencies ± standard deviation of CD107a expressing T cell subsets and B cells, and of intracellular INFy producing T cell subsets. Statistical tests were performed using multiple *t*-tests. Differences were considered highly significant with *p* values ≤ 0.005, and significant with *p* values ≤ 0.05. Abbreviations of subtypes are given in Table 1.

**Figure 4:**
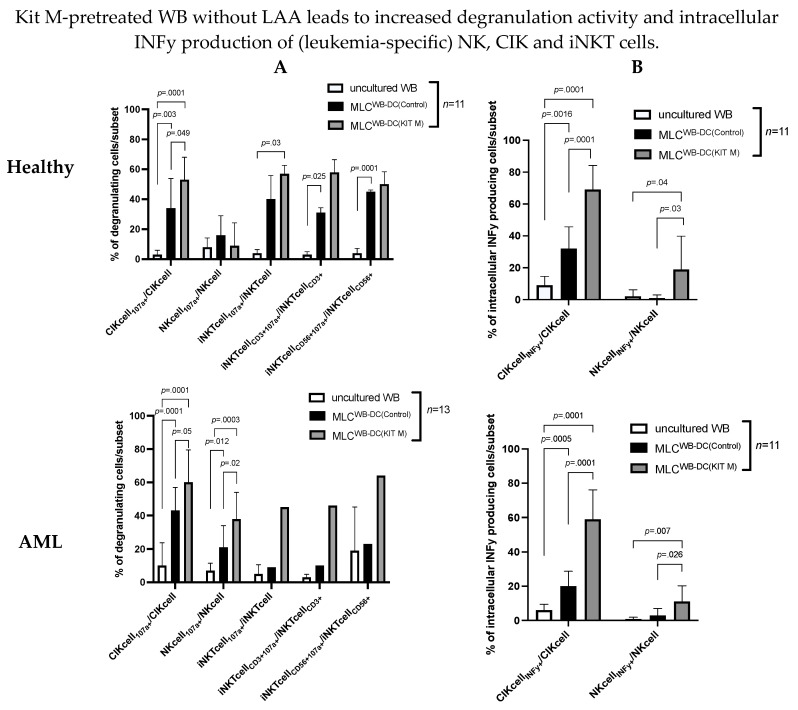
Stimulatory effect of Kit M-pretreated WB on (leukemia-specific) immunoreactive T cell subsets and B cells. Given are the degranulation activities (**A**) and intracellular INFy production of innate immune cells (**B**) of leukemic or healthy WB (uncultured WB), before and after culture of leukemic and healthy MLC pretreated with (MLC^WB-DC(KIT M)^) or without Kit M (MLC^WB-DC(Control)^), without LAA stimulation. Given are the average frequencies ± standard deviation of CD107a expressing NK, CIK and iNKT cell subsets, and of intracellular INFy producing NK and CIK cell subsets. Statistical tests were performed using multiple *t*-tests. Differences were considered highly significant with *p* values ≤ 0.005, and significant with *p* values ≤ 0.05. Abbreviations of subtypes are given in Table 1.

The degranulation activity of immune reactive cells was evaluated with a Deg assay in WB (uncultured WB) and after MLC (MLC^WB-DC(Control)^ and MLC^WB-DC(Kit M)^); the intracellular TNFa and INFy production was analyzed with an InCyt assay. Deg and InCyt assays were both conducted with/without stimulation with leukemia- associated antigens (LAA) (WT-1 and PRAME) for leukemic samples, or with/without stimulation with SEB for healthy samples (‘Stimulated’/‘Unstimulated’) (Figure 3 and Figure 4).

 

**Stimulation of (leukemia/antigen) specific immune cells was successful after healthy and leukemic MLC culture compared to uncultured WB**

In healthy and leukemic WB without SEB/LAA stimulation after MLC (vs. uncultured WB) (significantly), we found higher frequencies of antigen-specific degranulating and INFy-producing T cells (e.g., %CD3+_107a+/INFy+_/CD3+: Kit M: 32 ± 23; uncultured WB: 24 ± 15; *p* = 0.001 and %CD3+_107a+/INFy+_/CD3+: Control: 24 ± 15; uncultured WB: 6 ± 9; *p* = 0.01), degranulating non-naive T cells (T_non-naive107a+/INFy+_/T_non-naive_), effector memory and central memory T cell subsets (T_em/eff107a+/INFy+_/T_em/eff_) (T_cm107a+/INFy+_/T_cm_), degranulating B cells (Bcell_107a+_/Bcell) (Figure 3A,B), and several subtypes of degranulating innate immunity cells (CIKcell_107a+/INFy+/_CIKcell, NKcell_107a+/INFy+_/NKcell, iNKTcell_107a+_/iNKTcell) (Figure 4A,B).

(Data with vs. without SEB/LAA stimulation after MLC were similar (data not shown).

In summary, we conclude that IL-2 had a significant effect on the (antigen-specific) activation and differentiation of degranulating and INFy-producing innate and adaptive immunity cells after MLC compared to uncultured WB.

 

**Significantly higher frequencies of leukemia/antigen-specific, adaptive immune cells were found in MLC^WB-DC(Kit M)^ compared to MLC^WB-DC(Control)^ in healthy and AML samples**

In healthy samples without SEB stimulation, we found significantly higher frequencies of Bcell_107a+_/Bcell (%Bcell_107a+_/Bcell: Kit M: 32 ± 11; Control: 13 ± 4; *p* = 0.017) (Figure 3A ‘Healthy’), and (borderline) significantly higher frequencies of CD3+_INFy+_/CD3+, T_CD4+INFy+_/T_CD4+_, T_CD4−INFy+_/T_CD4−_, T_non-naive INFY+_/T_non-naive_ in MLC^WB-DC(Kit M)^ compared to MLC^WB-DC(Control)^ (e.g., %CD3+_INFy+_/CD3+: Kit M: 45 ± 19; Control: 24 ± 16; *p* = 0.025) (Figure 3B, ‘Healthy’). 

In leukemia samples without LAA stimulation, we found borderline significantly higher frequencies of T_non-naive107a+_/T_non-naive_ in MLC^WB-DC(Kit M)^ compared to MLC^WB-DC(Control)^ (%T_non-naive107a+_/T_non-naive_: Kit M: 48 ± 28; Control: 28 ± 17; *p* = 0.09) (Figure 3A ‘AML’). Significantly higher production of intracellular INFy in MLC^WB-DC(Kit M)^ compared to MLC^WB-DC(Control)^ was found in, e.g., CD3+_INFy+_, T_CD4−INFy+_, (%CD3+_INFy+_/CD3+: Kit M: 32 ± 15; Control: 14 ± 10; *p* = 0.006 and %T_CD4+INFy+_/T_CD4+_: Kit M: 50 ± 26; Control: 24 ± 17; *p* = 0.016 *(*Figure 3B ‘AML’).

We conclude that Kit M treatment (vs. no treatment) led to an increased degranulation activity and an increased production of intracellular INFy and TNFa in leukemic and healthy samples’ adaptive immune cells. 

 

**Significantly higher frequencies of leukemia/antigen-specific innate immune cells were found in MLC^WB-DC(Kit M)^ compared to MLC^WB-DC(Control)^ in healthy and AML samples**

In healthy samples, significantly higher frequencies of CIKcell_107+_/CIKcell (%CIKcell_107a+_/CIKcell: Kit M: 53 ± 15; Control: 34 ± 20; *p* = 0.049) (Figure 4A ‘Healthy’) and of CIKcell_INFy+_/CIKcell and NKcell_INFy+_/Nkcell were found after MLC^WB-DC(Kit M)^ compared to MLC^WB-DC(Control)^ (%CIKcell_INFy+_/CIKcell: Kit M: 69 ± 15; Control: 32 ± 14; *p* = 0.0001 and %Nkcell_INFy+_/Nkcell: Kit M: 19 ± 21; Control: 1 ± 2; *p* = 0.03) (Figure 4B ‘Healthy’). 

In leukemic samples, we found significantly higher frequencies of degranulating CIK and NK cells (%CIKcell_107a+_/CIKcell: Kit M: 60 ± 19; Control: 43 ± 14; *p* = 0.05 and %NKcell_107a+_/NKcell: Kit M: 38 ± 16; Control: 21 ± 13; *p* = 0.02) (Figure 4A ‘AML’) and of CIKcell_INFy+_/CIKcell and NKcell_INFy+_/NKcell in MLC^WB-DC(Kit M)^ compared to MLC^WB-DC(Control)^ (%CIKcell_INFy+_/CIKcell: Kit M: 59 ± 17; Control: 20 ± 9; *p* = 0.0001 and %NKcell_INFy+_/NKcell: Kit M: 11 ± 9; Control: 3 ± 4; *p* = 0.026) (Figure 4B ‘AML’).

We can conclude that Kit M-pretreated (vs. untreated) WB led to an increased degranulation activity and a higher production of intracellular INFy and TNFa of innate immune cells. 

 

**The antileukemic activity of T cell-enriched MLC is improved with Kit M-pretreated stimulated cells**

After co-culture of ‘effector cells’ (T cell-enriched MLC with Kit M-pretreated (vs. not pretreated) WB) with ‘target cells’ (thawed blast-containing MNC), we compared blast lysis of MLCWB-DC(Control) (‘Control’) vs. MLCWB-DC(Kit M) (‘Kit M’) using a cytotoxicity fluorolysis assay. Analyses were conducted after 3 or 24 h of incubation of the target with effector cells, and finally the better antileukemic effectivity after either 3 or 24 h was selected as the ‘best’ achieved lysis value. The lytic activity was calculated and defined as the frequencies of (un)viable target cells compared to a control [8,12]. 

**Figure 5:**
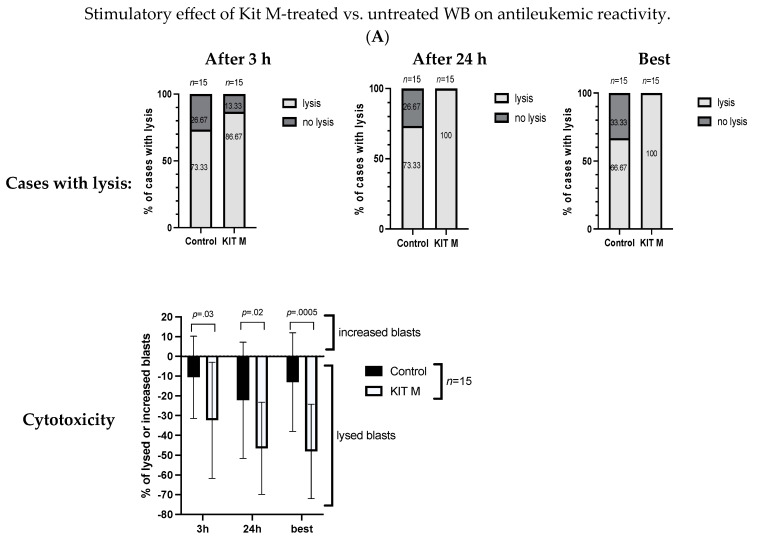

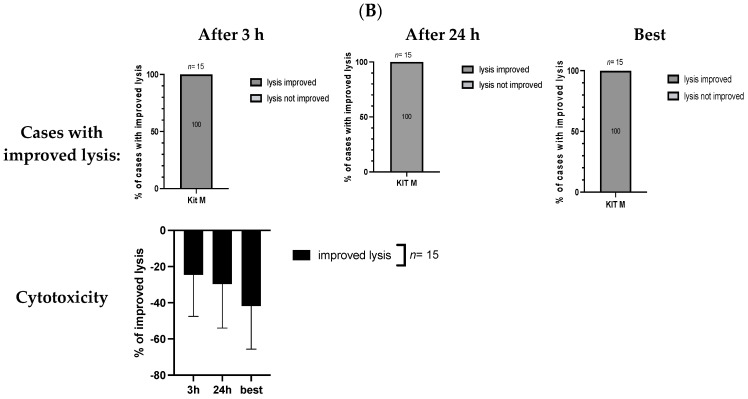
(**A**) Kit M-treated leukemic WB (compared to untreated WB) leads to stimulation of blast lytic activity. (**B**) Improved blast lysis with Kit M-pretreated WB compared to Control. Stimulatory effect of Kit M-pretreated (vs. untreated) WB on the cytotoxic activity after 3 h and 24 h of co-culture of immunoreactive cells (‘effector cells’) and blasts (‘target cells’). Given are the proportions of cases with blast lysis and the frequencies ± standard deviation of increased or lysed blasts after MLC^WB-DC(Kit M)^ (Kit M), compared to MLC^WB-DC(Control)^ (Control), after 3 h and 24 h, and the ‘best’ achieved blast lysis after 3 h or 24 h (**A**) and the proportions of cases with an improvement in blast lysis (or without an improvement in blast lysis), the frequencies ± standard deviation of improved (or not improved) lysed blasts after MLC^WB-DC(Kit M)^ in relation to MLC^WB-DC(Control)^ after 3 h and 24 h, and the ‘best’ achieved improvement in blast lysis (or without improvement in blast lysis) after 3 h or 24 h (**B**). Statistical tests were performed using a paired *t*-test. Differences were considered highly significant with *p* values ≤ 0.005, and significant with *p* values ≤ 0.05.

After 3 h, blast lysis was found in 86.67% of cases (13/15) after MLCWB-DC(Kit M) vs. 73.33% of cases (11/15) in the Control (MLCWB-DC(Control)). After 24 h, blast lysis was found in 100% of cases (15/15) after MLCWB-DC(Kit M) vs. 73.33% of cases (11/15) in Control (MLCWB-DC(Control)). After 3 h, the frequencies of lysed blasts after MLCWB-DC(Kit M) were significantly lower compared to MLCWB-DC(Control) (%lysed blasts: Kit M: −32.36 ± 29.34; Control: −10.62 ± 20.83; *p* = 0.03). After 24 h, we found significantly lower frequencies of lysed blasts after MLCWB-DC(Kit M) compared to MLCWC-DC(Control) (%lysed blasts: Kit M: −46.57 ± 23.32; Control: −22.19 ± 29.40; *p* = 0.02) (Figure 5A).

After 3 and 24 h of incubation, 100% (15/15) of cases showed improved lysis after MLCWB-DC(Kit M) compared to MLCWB-DC(Control). The proportions of cases with improved lysis after 3 h were −24.59 ± 22.93, and after 24 h were −29.62 ± 24.33 (Figure 5B). 

After selecting the ‘best’ achieved lysis value after 3 h and 24 h of incubation time, we found more cases with lysis after MLCWB-DC(Kit M) vs. MLCWB-DC(Control) (MLCWB-DC(Kit M): 100% vs. MLCWB-DC(Control): 66.67% cases of lysis). The frequencies of lysed blasts were significantly lower after MLCWB-DC(Kit M) compared to MLCWB-DC(Control) (%lysed blasts: Kit M: −48.07 ± 23.86; Control: −13.04 ± 25.02; *p* = 0.0005) (Figure 5A). We found improved blast lysis in 15/15 cases (100%) after MLCWB-DC(Kit M) compared to MLCWB-DC(Control), which only led to improved lysis of −41.82 ± 23.77% (Figure 5B).

In summary, Kit M pretreatment significantly improved blast lysis after MLC.

We can conclude that Kit M-pretreated (vs. untreated) leukemia and healthy WB gave rise to higher frequencies of mature (leukemia-derived) DC subtypes and (after MLC) of (leukemia) specific cells of several lines (innate and adaptive immunity cells, including memory cells), finally giving rise to blast-lysing cells.

### 3.2. Volatile Organic Compounds (VOC) Results

**Figure 6:**
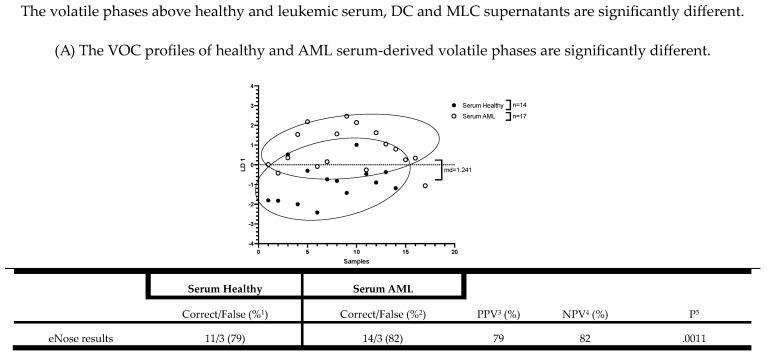

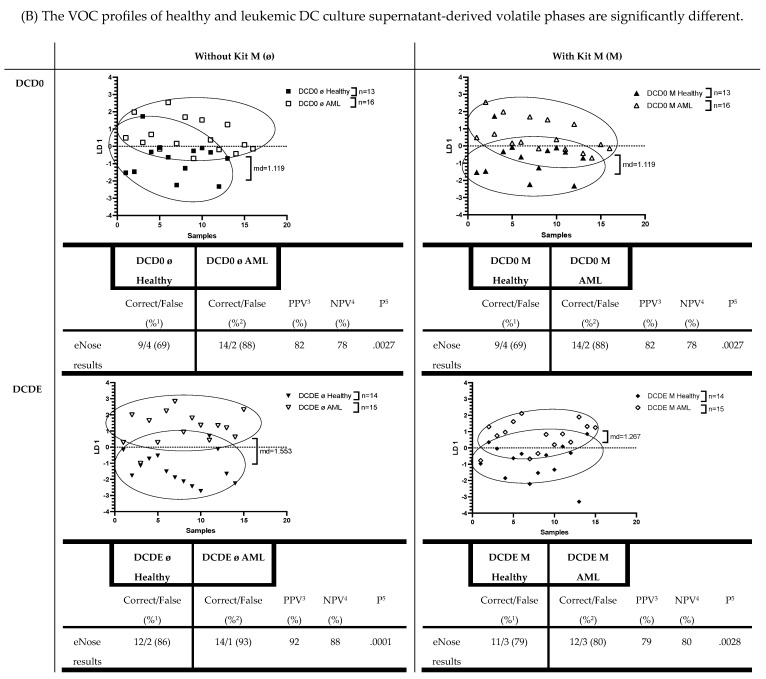

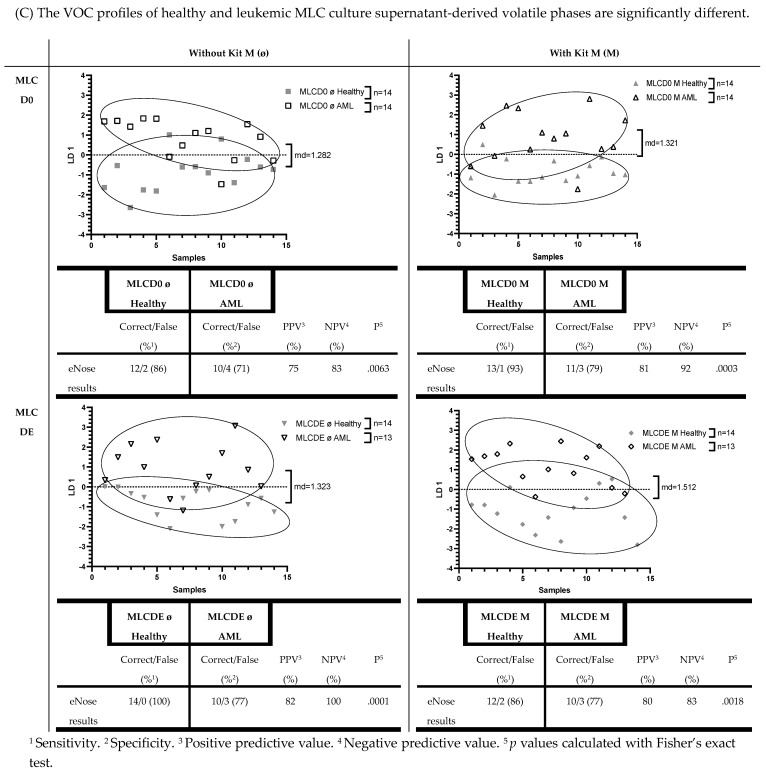
Significantly different eNose results between serum, DC culture, or MLC culture supernatant-derived VOCs using healthy or AML patients’ samples. Given are the absolute and graphically represented differences of volatile phases derived from healthy serum, DC culture supernatants (DCD0 ø, DCD0 M, DCDE ø, DCDE M), and MLC culture supernatants (MLCD0 ø, MLCD0 M, MLCDE ø, MLCDE M) compared to the corresponding leukemic volatile phases. Given underneath are the dimensionless LD 1 values and tables, presenting an overview of the sensitivity, specificity, negative and positive predictive values. Statistical tests were performed using Fisher’s exact test: Differences were considered significant with *p* values ≤ 0.05. Abbreviations of analyzed samples are given in Table 2.

**Figure 7:**
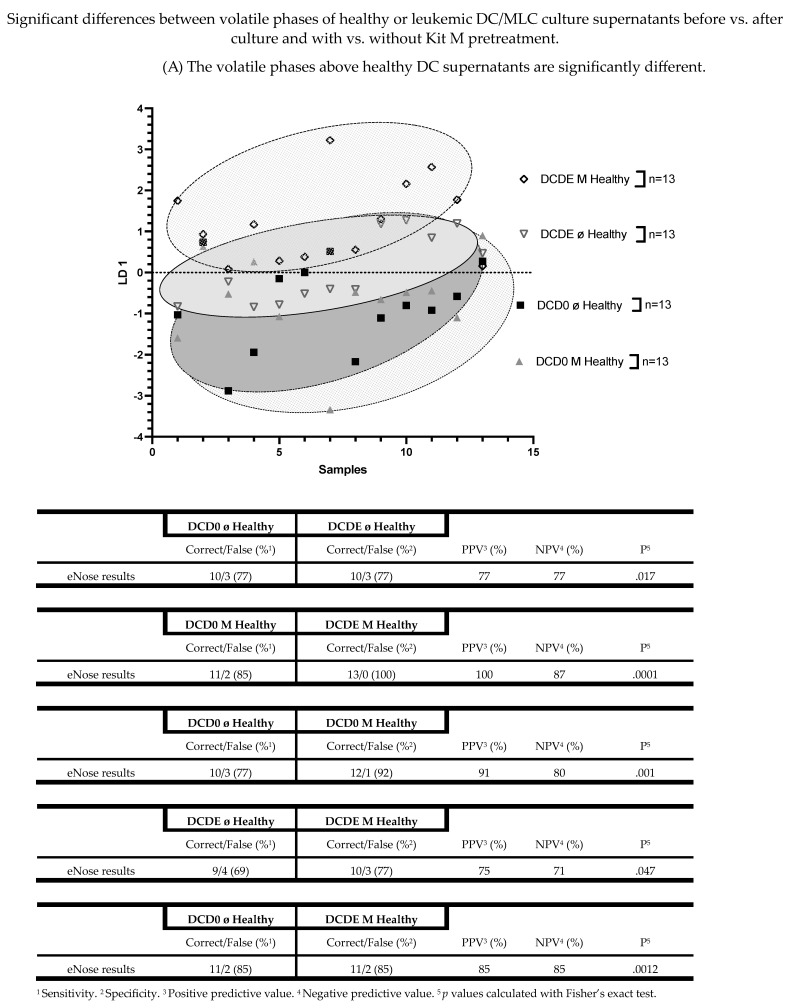

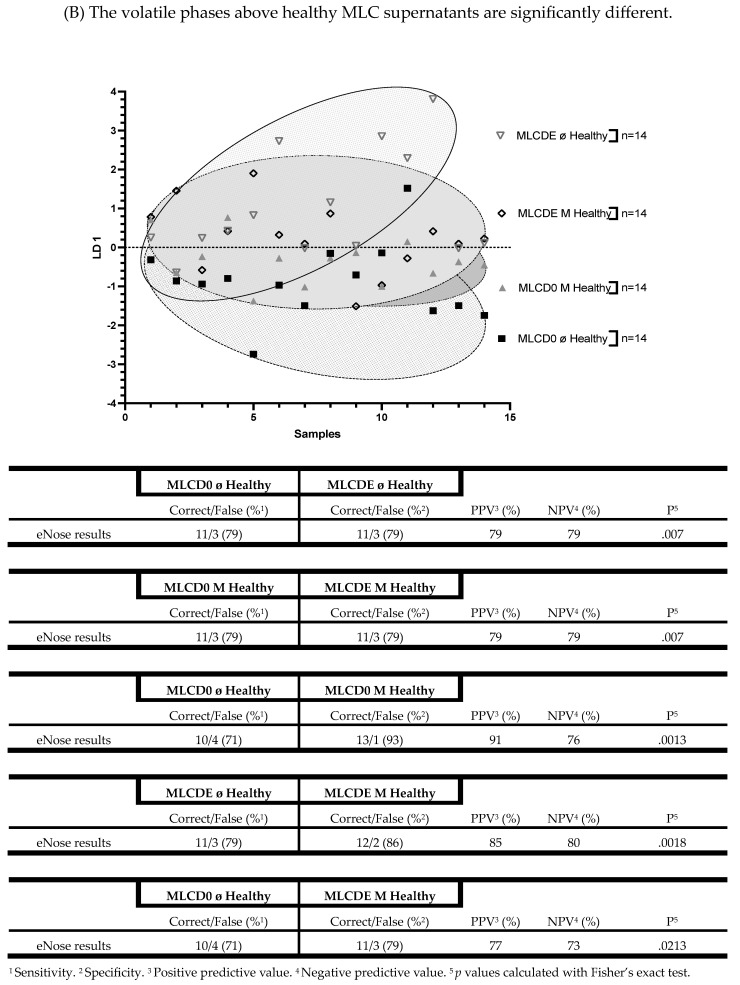

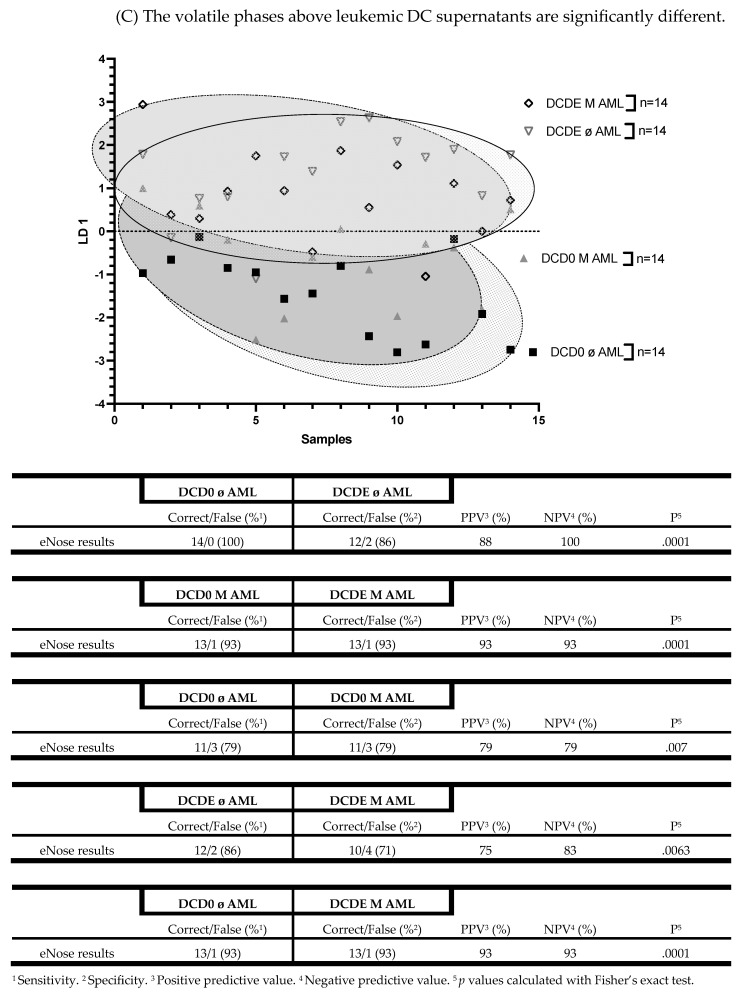

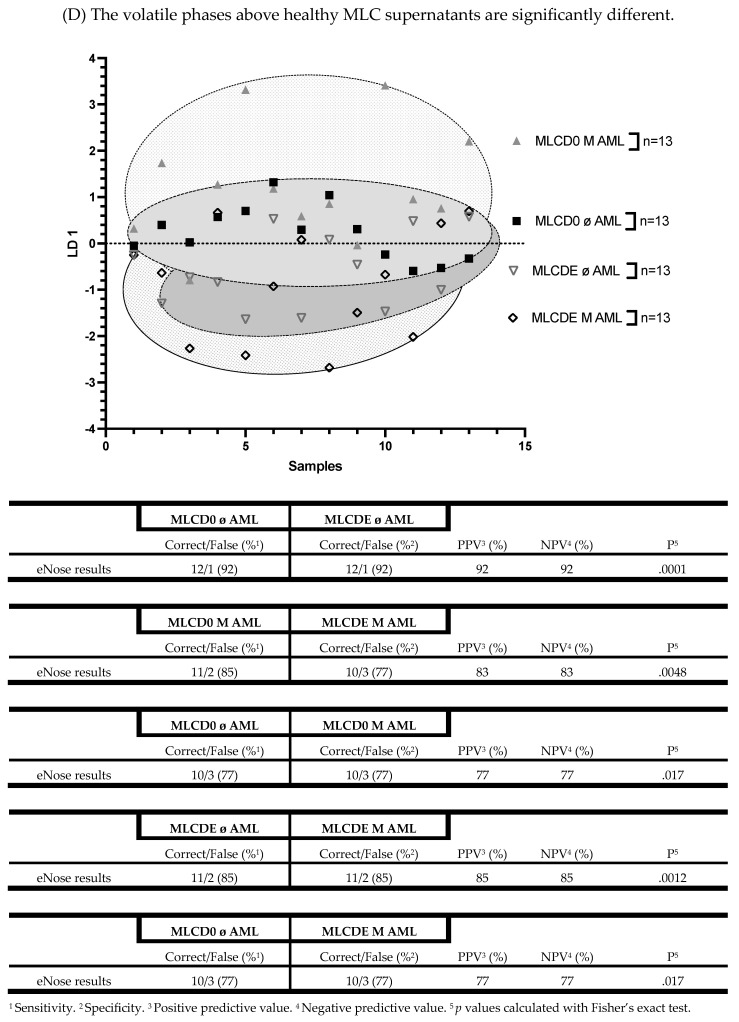
Shown are the graphically represented differences of volatile phases derived from either healthy and leukemic DC culture supernatants (DCD0 ø, DCD0 M, DCDE ø, DCDE M) or healthy and leukemic MLC culture supernatants, comparing before vs. after culture and with vs. without Kit M pretreatment. Given underneath are the dimensionless LD 1 values and tables, presenting an overview of the sensitivity, specificity, negative and positive predictive values. Statistical tests were performed using the Fisher’s exact test. Differences were considered significant with *p* values ≤ 0.05. Abbreviations of analyzed samples are given in Table 2.

**Figure 8:**
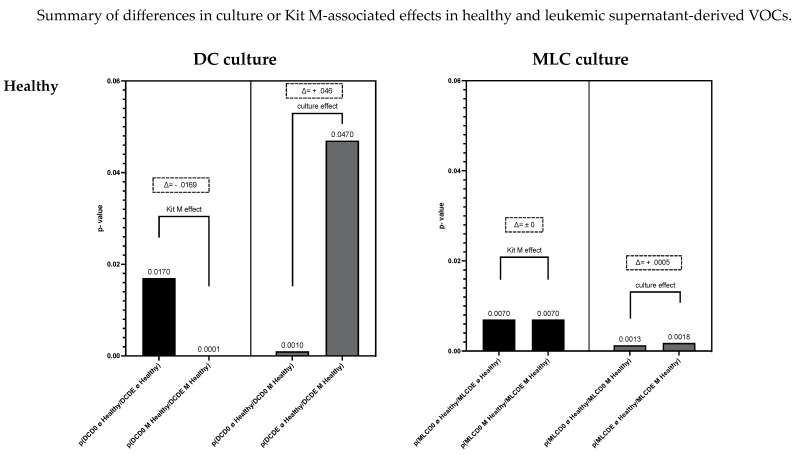

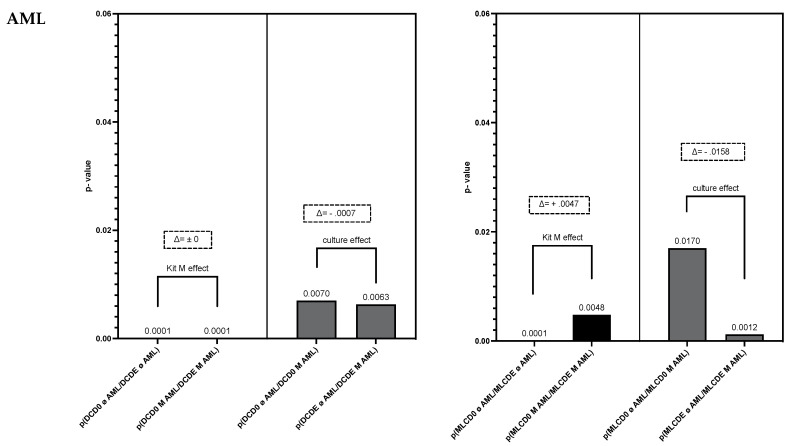
Given are the summarized VOC results, focusing on culture- or Kit M-mediated effects on the identification with eNose of VOC profiles derived from healthy or leukemic DC or MLC culture supernatants. The X-axis shows *p* values from comparisons from Figure 7A–D. Statistical tests were performed using Fisher’s exact test. Differences were considered significant with *p* values ≤ 0.05. Differences between *p* values are shown with Δ. Differences were considered significant with Δ ≥ I0.001I. Abbreviations of analyzed samples are given in Table 2.

Serum supernatants and cell culture supernatants were obtained from healthy and AML samples’ serum, DC, and MLC cultures. As a source for VOC analyses conducted using eNose, we used volatile phases above healthy and leukemic serum supernatants (‘Serum’), DCD0 ø/MLCD0 ø, DCDE ø/MLCDE ø (DC/MLC culture without Kit M treatment), DCD0 M/MLCD0 M, and DCDE M/MLCDE M (DC/MLC culture pretreated with Kit M), as described in the section *DC culture* or *MLC culture*. An overview of the collected supernatants of every proband is given in Table 2, and of the VOC experiments in Scheme 2. After measuring, various comparisons were calculated using linear discriminant analyses (LD), and the smellprints are graphically shown in two-dimensional principal component analysis (PCA) plots (Figure 6, Figure 7 and Figure 8). The sensitivity, specificity, Mahalanobis distance (md), negative and positive predictive values, and the *p*-value calculated by Fishers’ exact test gave information about the significant discrimination.

 

**The VOC profiles of the healthy and AML serum-derived volatile phases are significantly different**

Using 14 healthy and 17 AML patients’ serum supernatants, the eNose showed a significant differentiation (%sensitivity: 79; %specificity: 82; *p* = 0.0011). Detailed information about the serum results obtained with the eNose are given in Figure 6A.

### 3.3. The VOC Profiles of Uncultured and Cultured Healthy and Leukemic DC Culture Supernatants (with/without Kit M Pretreatment) of Derived Volatile Phases Are Significantly Different

We differentiated VOCs in supernatants from healthy vs. leukemic cultures. In healthy DC culture supernatant-derived volatile phases, we found significantly different VOC results compared to leukemic DC culture supernatants. Healthy donors’ DCD0 ø and DCD0 M achieved a sensitivity of 69%, and AML patients’ DCD0 ø and DCD0 M a specificity of 88% (healthy vs. leukemic DCD0 ø and healthy vs. leukemic DCD0 M: *p* = 0.0027). A less significant difference was found in healthy and leukemic DCDE M (%sensitivity: 79; %specificity: 80; *p* = 0.0028). A more significant difference was found in healthy and AML patients’ DCDE ø (%sensitivity: 86; %specificity: 93; *p* = 0.0001). Graphs and more details are shown in Figure 6B).

### 3.4. The VOC Profiles of Uncultured and Cultured Healthy and Leukemic MLC Culture Supernatants (with/without Kit M Pretreatment) of Derived Volatile Phases Are Significantly Different

eNose analyses of healthy and AML samples’ MLC culture supernatants achieved significantly different results. With a sensitivity of 86% and a specificity of 71%, the eNose could separate healthy MLCD0 ø from leukemic MLCD0 ø (*p* = 0.0063). Compared to AML patients’ supernatant, we found significant differences in healthy MLCD0 M (%sensitivity: 93; %specificity: 79; *p* = 0.0003), in healthy MLCDE ø (%sensitivity: 100; %specificity: 77; *p* = 0.0001), and in healthy MLCDE M (%sensitivity: 86; %specificity: 77; *p* = 0.0018) (Figure 6C)). 

In summary, we conclude that the eNose, by using LD analyses, could significantly differentiate volatile phases above both healthy and leukemic serum supernatants, and healthy and leukemic DC and MLC culture supernatants (both cultured and uncultured DC/MLC) in a direct comparison. Furthermore, we conclude that the eNose yielded significantly different results between healthy and leukemic DC and MLC supernatants, whether pretreated or not with Kit M.

### 3.5. The Volatile Phases above Healthy DC Supernatants Are Significantly Different

We differentiated VOCs from culture supernatants before vs. after culture and with vs. without Kit M pretreatment in healthy or in AML VOC samples. We found significantly different volatile phases in several healthy DC supernatants (DCD0 ø vs. DCD0 M vs. DCDE ø vs. DCDE M). Healthy DCD0 ø compared to healthy DCDE ø showed a significant difference in VOC analyses (%sensitivity: 77; %specificity: 77; *p* = 0.017). Moreover, healthy DCD0 M compared to healthy DCDE M could be distinguished most significantly (%sensitivity: 85; %specificity: 100; *p* = 0.0001). The eNose achieved a sensitivity of 77% and a specificity of 92% in healthy DCD0 ø compared to DCD0 M (*p* = 0.001). Furthermore, analyses of healthy DCDE ø and healthy DCDE M yielded a significant difference (%sensitivity: 69; %specificity: 77; *p* = 0.047). Healthy DCD0 ø supernatants compared to DCDE M supernatants were identified by eNose, with a sensitivity of 85% and a specificity of 85% (*p* = 0.0012) (Figure 7A). 

### 3.6. The Volatile Phases above Healthy MLC Supernatants Are Significantly Different

The eNose could significantly differentiate between the volatile phases of healthy MLC culture supernatants, both before vs. after culture and with vs. without Kit M pretreatment (MLCD0 ø vs. MLCD0 M vs. MLCDE ø vs. MLCDE M). We found a significant difference between healthy MLCD0 ø and healthy MLCDE ø (%sensitivity: 79; %specificity: 79; *p* = 0.007). With the same results, we could separate healthy MLCD0 M from healthy MLCDE M (%sensitivity: 79; %specificity: 79; *p* = 0.007).

The eNose could also significantly distinguish healthy MLCD0 ø from healthy MLCD0 M (%sensitivity: 71; %specificity: 93; *p* = 0.0013). Moreover, we found significantly different VOC results in healthy MLCDE ø compared to healthy MLCDE M (%sensitivity: 79; %specificity: 86; *p* = 0.0018). A less significant difference was achieved in a VOC analysis with healthy MLCD0 ø and healthy MLCDE M (%sensitivity: 71; %specificity: 79; *p* = 0.0213) (Figure 7B).

### 3.7. The Volatile Phases above Leukemic DC Culture Supernatants Are Significantly Different

For analyzing the eNose’s ability to distinguish several AML patients’ DC culture supernatants (before vs. after culture and with vs. without Kit M pretreatment), we used 14 AML supernatants. We found significantly different VOC results in leukemic DCD0 ø compared to leukemic DCDE ø (%sensitivity: 100; %specificity: 86; *p* = 0.0001). We investigated the difference between leukemic DCD0 M and leukemic DCDE M, as well as the difference between leukemic DCD0 ø and leukemic DCDE M. In both, we found similar significantly different results (%sensitivity: 93; %specificity: 93; *p* = 0.0001). Furthermore, the eNose could significantly distinguish leukemic DCD0 ø from leukemic DCD0 M (%sensitivity: 79; %specificity: 79; *p* = 0.007). In addition, we investigated the LD analysis of leukemic DCDE ø and leukemic DCDE M. The eNose could correctly identify the leukemic DCDE ø with a sensitivity of 86%, and the leukemic DCDE M with a specificity of 71% (*p* = 0.0063) (Figure 7C).

### 3.8. The Volatile Phases above Leukemic MLC Culture Supernatants Are Significantly Different

The eNose could significantly differentiate between the volatile phases of leukemic MLC culture supernatants before vs. after culture and with vs. without Kit M pretreatment. We found significantly different volatile phases in 13 several leukemic MLC supernatants (MLCD0 ø vs. MLCD0 M vs. MLCDE ø vs. MLCDE M). Leukemic MLCD0 ø showed significantly different VOC results compared to leukemic MLCDE ø (%sensitivity: 92; %specificity: 92; *p* = 0.0001). The comparison of AML patients’ MLCD0 M and MLCDE M yielded a sensitivity of 85% and a specificity of 77%, which led to a significant difference in their VOC results (*p* = 0.0048). In addition, the eNose significantly distinguished leukemic MLCD0 ø from leukemic MLCD0 M (%sensitivity: 77; %specificity: 77; *p* = 0.017). With the same effectiveness, the eNose could separate leukemic MLCD0 ø from leukemic MLCDE M (%sensitivity: 77; %specificity: 77; *p* = 0.017). More significant results could be achieved in a comparison of leukemic MLCDE ø with leukemic MLCDE M (%sensitivity: 85; %specificity: 85; *p* = 0.0012) (Figure 7D).

Using LD analyses, we conclude that the eNose could significantly distinguish volatile phases above healthy DC (DCD0 ø vs. DCD0 M or DCDE ø vs. DCDE M) and healthy MLC (MLCD0 ø vs. MLCD0 M or MLCDE ø vs. MLCDE M) culture supernatants, and above several AML patients’ DC (DCD0 ø or DCD0 M vs. DCDE ø vs. DCDE M) and leukemic MLC (MLCD0 ø vs. MLCD0 M or MLCDE ø vs. MLCDE M) culture supernatants.

 

**Summary of differences in culture and Kit M-associated effects on healthy or leukemic supernatant-derived VOCs**

Figure 8 gives an overview of healthy and leukemic (with or without Kit M-pretreated) cultures (DC/MLC)-derived VOCs, and the influence of culture or Kit M-associated effects. In healthy DC culture supernatant-derived VOCs, we found a Kit M-associated effect (Δ*p* = −0.0169) and a high culture-associated effect (Δ*p* = 0.046) (Figure 8; upper left). In healthy MLC culture supernatant-derived VOCs, we found no Kit M effect (Δ*p* = 0) and a small culture effect (Δ*p* = 0.0005) (Figure 8; upper right). In AML patients’ DC culture supernatant-derived VOCs, we found (in contrast to healthy samples) no Kit M effect (Δ*p* = 0) and a small culture effect (Δ*p* = −0.0007) (Figure 8; lower left). Moreover, (compared to healthy samples) we found significant Kit M-mediated differences in leukemic MLC culture supernatant-derived VOCs (Δ*p* = 0.0047) alongside high culture effects (Δ*p* = −0.0158) (Figure 8; lower right).

Following our results, we can summarize that the eNose could significantly distinguish between healthy and leukemia patients’ serum, DC (DCD0 ø, DCD0 M, DCDE ø, DCDE M) and MLC (MLCD0 ø, MLCD0 M, MLCDE ø, MLCDE M) culture supernatant-derived volatile phases. Moreover, the eNose could significantly separate several supernatant (with vs. without Kit M treatment, cultured vs. uncultured)-derived VOCs within subgroups (healthy DC or leukemic DC or healthy MLC or leukemic MLC supernatants). Furthermore, the eNose could indicate a clear Kit M-associated effect in healthy cultures (but not in AML-DC cultures), and clear Kit M-mediated effects in leukemic MLC cultures.

## 4. Discussion

### 4.1. DC Based Immunotherapy

We can confirm with preliminary data that DC/DC_leu_ can be generated in Kit M-pretreated (vs. not pretreated) healthy patients’ WB samples without inducing blast/monocyte proliferation [8,12,22,46,47]. Tendentially, more (mature) DC could be generated from healthy samples’ WB, probably due to partly impaired DC generation from patients’ WB due to the inherent immune suppressive effect of leukemia [15]. Moreover, we could confirm the immune cell activation of Kit M-pretreated WB after MLC; cells of the adaptive as well as of the innate immune system were activated and had increased frequencies vs. Control. Increased frequencies of immune cells after MLC compared to uncultured MLC are due to the influence of IL-2, as already previously stated [12,15,48]. We confirm with preliminary data that Kit M-treated (vs. untreated) WB leads to (antigen-specific) activation of immune cells and increased antileukemic activity (or ‘cytotoxicity’) in cases with AML after MLC [12]. This antileukemic effect can be ascribed to the different killing mechanisms of Kit M-mediated immunoreactive cells, i.e., the faster perforin/granzyme pathways and the slower Fas/FasL pathway [12,49].

### 4.2. VOC Based AML Monitoring

To monitor AML patients’ disease or antileukemic immunoreactions, analyses of different cellular, humoral, and soluble factors are important [12,15,22,24,25,26]. VOCs released by leukemic or immune cells’ processes and measured directly by the eNose might be an important tool to monitor biomarkers related to the disease, as previously shown in VOC studies with other diseases [31,36]. Moreover, quantitative and qualitative evaluations of VOCs (collected above cell culture supernatants) were shown to be technically possible, and could show correlations with subtypes of the underlying disease [38]. 

### 4.3. Strengths and Limitations of VOC Analyses

An electronic nose such as the one used here utilizes a standardized approach. An important requirement is that its sensors work correctly, and the 32 sensors of the eNose indicate correct reference ranges. Moreover, sensors must be trained with a training set before the start of measurements and must be regularly calibrated. In contrast to other methods, such as ion mobility spectrometry or mass spectrometry, identification of the individual component in a mixture is usually not possible here or is only possible under very restricted conditions [50]. Consequently, this method has the disadvantage that identification of individual analytes in a complex mixture, such as exhaled air, is usually not possible. Conversely, it provides an overall picture of all volatile compounds present in the breath, including those that were not originally the focus of interest but could be a previously unrecognized indicator of (systemic) disease. The principle of the evaluation is based on pattern recognition, so the identification of individual substances is not necessary for classification applications. One of the big advantages of this method is its mobility, and its fast measurement process, which takes only 30 s. In previous studies, it was shown that samples of exhaled air as well as liquid samples could be used as a medium, whereas solid, non-organic materials could not. The Cyranose 320^®^ polymer sensor enables investigations in the ppm range [mL/m^3^]. Its 32 sensors have a variation of coefficient of 2–10%. The addition of all 32 signals and the determination of the precision of the total signal finally resulted in a mean coefficient of variation of 4.64%. Digitally sensed VOCs using an electronic nose have the potential to become a rapid, immediate, and non-invasive diagnostic tool. With the advent of inexpensive, environmentally friendly, and biocompatible sensor systems, health monitoring using VOCs may transform laborious or invasive procedures that currently can only be used in facilities specialized for this purpose into a technology that can be used anywhere and anytime by individuals.

### 4.4. VOC Differentiation of Healthy vs. Leukemic Serum, DC- and MLC-Cultures as a New Refined, Clinical Monitoring Tool

Analyses of different cellular/humoral, soluble factors [8,15,21,22,23,24] or circulating vesicles (e.g., extracellular vesicles (EVs) [26,27]) were tested with respect to a refined monitoring of immunological or tumor-associated processes. VOC differentiation of healthy and leukemic serum or culture samples might be a new strategy for screening recurrence and quantifying the tumor load of diseased patients. Our study demonstrates that an eNose could significantly distinguish healthy VOCs from leukemic VOCs derived from serum, DC- and MLC-culture supernatants. This was true for both healthy and leukemic Kit M-pretreated and non-pretreated cell culture supernatants analyzed using the eNose (Figure 6). Differences in VOCs could have cell biological/immunological causes; AML blasts produce (in contrast to healthy samples) specific mediators and factors (e.g., IL-1-b, IL-6 and angioregulatory factors) to stimulate their proliferation [50,51]. Moreover, the differing frequencies of cell compositions in healthy and leukemic cultures secreting variant factors into the supernatant might lead to different VOCs (Figure 1 and Figure 2); for example, DC_mat_ secrete different exosomes compared to immature DCs (there are higher frequencies of DC_mat_ subsets in healthy vs. leukemic samples). Exosomes from mature DCs (compared to immature DCs) can be enriched with MHC class II, B7.2, and ICAM-1, and depleted in MFG-E8 [52].

### 4.5. IL-2-Associated Effects on Differences between Uncultured and Cultured MLC-VOCs

A culture effect could be detected by the eNose in leukemic MLC culture supernatant-derived VOCs. VOCs collected before culture were significantly different compared to after culture (leukemic MLC: Δ*p* = −0.0158) (Figure 8). These VOC results can probably be explained by the IL-2 effect. Following our cell biological results, IL-2 had a stimulatory effect on the immune cells and a significant effect on the provision and activation of (antigen-specific) immune cells (compared to uncultured cells). However, this effect could only be measured in leukemic MLC culture supernatant-derived VOCs.

### 4.6. Kit M-Associated Effects on DC- and MLC-VOCs

We could deduce the following culture and Kit M-related effects detectable by VOC profiles in healthy and AML cultures:

1. With respect to culture effects, we found significant differences in the VOC profiles of healthy DC culture supernatants (independent of the addition of Kit M), whereas the culture effects of AML samples in the same settings were not different. These findings might be explained by the different compositions of DC culture supernatants in healthy vs. AML DC culture supernatants; healthy samples contain higher frequencies of ‘healthy cells’, and AML samples contain high frequencies of blasts. After culture, different releases of VOCs in the different settings might explain the good differentiation of healthy DC supernatant VOCs, but not AML DC culture supernatant VOCs.
2. With respect to Kit M-mediated effects, we found significant differences in the VOC profiles of healthy DC culture supernatants when comparing Kit M-pretreated vs. non-pretreated samples, whereas the culture effects of AML samples in the same settings were not different. These findings might be explained by different DC compositions in healthy vs. AML DC culture supernatants; healthy samples yield higher frequencies of mature monocyte-derived DCs, and AML samples yield (in addition) leukemia-derived DCs and blasts, which may proliferate/differentiate and produce leukemia-associated VOCs.
3. Moreover, AML patients’ DC supernatants might contain traces of drug (derivates) after chemotherapy and antibiotic/antimycotic therapy, which could be responsible for alternated VOC profiles compared to healthy DC culture supernatant-derived VOCs.
4. With respect to culture effects, we found no significant differences in the VOC profiles of healthy MLC supernatants (independent of Kit M addition), whereas the culture effects of AML samples in the same settings were significantly different. These findings might be explained by different MLC-related supernatants in healthy vs. AML MLC supernatants (e.g., higher frequencies of ‘healthy immune cells’ in healthy samples, and high frequencies of blasts in AML samples).
5. With respect to Kit M-mediated effects, we did not find significant differences in the VOC profiles of healthy MLC supernatants when comparing Kit M-pretreated vs. non-pretreated samples, whereas the culture effects of AML samples in the same settings were significantly different. These findings might be explained by the different compositions of immune cells in AML vs. healthy cells; the activation of leukemia-specific immune reactive cells in AML cases might yield significantly different VOCs under the influence of Kit M vs. Control.

This means that culture alone gives rise to other changes in healthy and AML VOC profiles; the presence of Kit M changes the setting and produces different results in AML and healthy VOC samples. 

## 5. Conclusions

We have shown that the role and metabolic influences of drugs of different cellular compositions with respect to qualitative/quantitative differences in VOC release profiles are complex, although our chosen technology yielded differences in different settings. Other strategies or more standardized settings might contribute to more refined VOC-based monitoring strategies in the future. According to our results, eNose analyses might then yield a prospective option for deriving a VOC-based disease profiling strategy using serum or cell culture supernatants from patients with leukemia. 

Due to rapid sample collection and analysis, the present study shows good reproducibility of data. It could therefore be recommended to include VOC analyses as an additional component to monitor the course of disease, and potentially to guide therapy-related decisions.

## Figures and Tables

**Table 1:** Cells and cell subsets as evaluated by flow cytometry.

		Name of Subgroups	Abbreviation of Subgroups	Surface Marker	Referred to	Abbreviation	Reference
	Blast cells	Blasts	BLA	BLA, e.g., CD34+, CD117+	WB (whole blood)	BLA/WB	[16]
		Proliferating blasts	BLA_prol-CD71_	BLA+DC-CD71+	BLA	BLA_prol-CD71_/BLA	[17]
		Proliferating blasts	BLA_prol-IPO38_	BLA+DC-IPO38+	BLA	BLA_prol-IPO38_/BLA	[6]
	Monocytoid cells	CD14+ monocytes	Mon	CD14+	WB	Mon/WB	[18]
		Proliferating CD14+ monocytes	Mon_prol-CD71_	CD14+DC-CD71+	CD14+	Mon_prol-CD71_/Mon	[9]
		Proliferating CD14+ monocytes	Mon_prol-IPO38_	CD14+DC-IPO38+	CD14+	Mon_prol-IPO38_/Mon	[9]
	Dendritic cells	Dendritic cells	DC	DC+, e.g., CD80+, CD206+	WB	DC/WB	[16]
		Leukemia-derived DC	DC_leu_	DC+BLA+	WB or DC or BLA	DC_leu_/WB DC_leu_/BLA	[16]
		Mature migratory DC	DC_mat_	DC+CD197+	WB or DC	DC_mat_/WB DC_mat_/DC	[18]
		Mature migratory DC_leu_	DC_leu-mat_	DC+BLA+CD197+	WB or DC or DC_leu_ oder DC_mat_ or BLA	DC_leu-mat_/WB DC_leu-mat_/DC DC_leu-mat_/BLA	[18]
	B lymphocytes	CD19+ B cells	Bcell	CD19+	lymphocytes	Bcell/cells	[19]
	T lymphocytes	CD3+ pan T cells	CD3+	CD3+	lymphocytes	CD3+/cells	[8]
		CD4+ coexpressing T cells	T_CD4+_	CD3+CD4+	CD3+	T_CD4+_/CD3+	[8]
		CD8+ coexpressing T cells	T_CD4−_	CD3+CD4−	CD3+	T_CD4−_/CD3+	[8]
		Naive T cells	T_naive_ T_naiveCD4+_ T_naiveCD4−_	CD3+CD45RO− CD3+CD45RO-CD4+ CD3+CD45RO-CD4−	CD3+ or T_CD4+_ or T_CD4−_	T_naive_/CD3+ T_naiveCD4+_/T_CD4+_ T_naiveCD4−_/T_CD4−_	[12]
Adaptive immune system		Non-naive T cells	T_non-naive_ T_non-naiveCD4+_ T_non-naiveCD4−_	CD3+CD45RO+ CD3+CD45RO+CD4+ CD3+CD45RO+CD4−	CD3+ or T_CD4+_ or T_CD4−_	T_non-naive_/CD3+ T_non-naiveCD4+_/T_CD4+_ T_non-naiveCD4−_/T_CD4−_	[12]
		Central memory T cells	T_cm_ T_cmCD4+_ T_cmCD4−_	CD3+CD45RO+CD197+ CD3+CD45RO+CD197+CD4+ CD3+CD45RO+CD197+CD4−	CD3+ or T_CD4+_ or T_CD4−_	T_cm_/CD3+ T_cmCD4+_/T_CD4+_ T_cmCD4−_/T_CD4−_	[12]
		Effector memory T cells	T_em/eff_ _Tem/effCD4+_ _Tem/effCD4−_	CD3+CD45RO+CD197− CD3+CD45RO+CD197-CD4+ CD3+CD45RO+CD197-CD4−	CD3+ or T_CD4+_ or T_CD4−_	T_em/eff_/CD3+ T_em/effCD4+_/T_CD4+_ T_em/effCD4−_/T_CD4−_	[12]
		Proliferating T cells, early	T_prol-early_	CD3+CD69+	CD3+	T_prol-early_/CD3+	[12]
		Proliferating T cells, late	T_prol-late_	CD3+CD71+	CD3+	T_prol-late_/CD3+	[12]
Leukemia-specific cells							
	B lymphocyte cells	CD19+ B cells_leu_	Bcell_107a+_	CD19+CD107a+	Bcell	Bcell_107a+_/Bcell	
	T lymphocyte cells	CD3+ pan T cells_leu_	CD3+107a+ CD3+INFy+ CD3+TNFa+ CD3+INFy+TNFa+	CD3+CD107a+ CD3+INFy+ CD3+TNFa+ CD3+INFy+TNFa+	CD3+ CD3+ CD3+ CD3+	CD3+_107a+_/CD3+ CD3+_INFy+_/CD3+ CD3+_TNFa+_/CD3+ CD3+_INFy+TNFa+_/CD3+	[20] [12]
		CD4+ coexpressing T cells_leu_	T_CD4+107a+_ T_CD4+INFy+_ T_CD4+TNFa+_ T_CD4+INFy+TNFa+_	CD3+CD4+CD107a+ CD3+CD4+INFy+ CD3+CD4+TNFa+ CD3+CD4+INFy+TNFa+	T_CD4+_ T_CD4+_ T_CD4+_ T_CD4+_	T_CD4+107a+_/T_CD4+_ _TCD4+INFy+_/T_CD4+_ T_CD4+TNFa+_/T_CD4+_ T_CD4+INFy+TNFa+_/T_CD4+_	[20] [12]
Adaptive immune system		CD8+ coexpressing T cells_leu_	T_CD4107a+_ T_CD4−INFy+_ T_CD4−TNFa+_ T_CD4−INFy+TNFa+_	CD3+CD4−CD107a+ CD3+CD4−INFy+ CD3+CD4−TNFa+ CD3+CD4−INFy+TNFa+	T_CD4−_ T_CD4−_ T_CD4−_ T_CD4−_	T_CD4−107a+_/T_CD4−_ _TCD4−INFy+_/T_CD4−_ T_CD4−TNFa+_/T_CD4−_ T_CD4−INFy+TNFa+_/T_CD4−_	[20] [12]
		Naive T cells_leu_	T_naive107a+_ T_naive INFy+_	CD3+CD45RO-CD107a+ CD3+CD45-INFy+	T_naive_ T_naive_	T_naive107a+_/T_naive_ T_naive INFy+/_T_naive_	[20] [12]
		Non-naive T cells_leu_	T_non-naive107a+_ T_non-naive INFy+_	CD3+CD45RO+CD107a+ CD3+CD45RO+INFy+	T_non-naïve_ T_non-naive_	T_non-naive107a+_/T_non-naive_ T_non-naïve INFy+/_T_non-naive_	[20] [12]
		Central memory T cells_leu_	T_cm107a+_ T_cm INFy+_	CD3+CD45RO+CD197+CD107a+ CD3+CD45RO+CD197+INFy+	T_cm_ T_cm_	T_cm107a+_/T_cm_ T_cm INFy+/_T_cm_	[20] [12]
		Effector memory T cells_leu_	T_em/eff107a+_ T_em/eff INFy+_	CD3+CD45RO+CD197-CD107a+ CD3+CD45Ro+CD197+INFy+	T_em/eff_ T_em/eff_	T_em/eff107a+_/T_em/eff_ T_em/eff INFy+_/T_em/eff_	[20] [12]
Innate	Cytokine-induced killer cells	CD3+CD56+ CIK cells_leu_	CIKcell_107a+_ CIKcell_INFy+_ CIKcell_TNFa+_ CIKcell_INFy+TNFa+_	CD3+CD56+CD107a+ CD3+CD56+INFy+ CD3+CD56+TNFa+ CD3+CD56+INFy+TNFa+	CIKcell CIKcell CIKcell CIKcell	CIKcell_107a+_/CIKcell CIKcell_INFy+_/CIKcell CIKcell_TNFa+_/CIKcell CIKcell_INFy+TNFa+/_CIKcell	[20] [12]
Immune system	Natural killer cells	CD3-CD56+ NK cells_leu_	NKcell_107a+_ NKcell_INFy+_ NKcell_TNFa+_ NKcell_INFy+TNFa+_	CD3-CD56+CD107a+ CD3-CD56+INFy+ CD3-CD56+TNFa+ CD3-CD56+INFy+TNFa+	NKcell NKcell NKcell NKcell	NKcell_107a+_/NKcell NKcell_INFy+_/NKcell NKcell_TNFa+_/NKcell NKcell_INFy+TNFa+_/NKcell	[20] [12]
	Invariant natural killer T cells	6B11+ iNKT cells_leu_	iNKTcell_107+_	6B11+CD107a+	NKcell	iNKTcell_107a+/_NKcell	[20]
		CD3+ coexpressing 6B11+ iNKT cells_leu_	iNKTcell_CD3+107a+_	6B11+CD3+CD107a+	NKcell	iNKTcell_CD3+107a+_ /NKcell	[20]
		CD56+ coexpressing 6B11+ iNKT cells_leu_	iNKTcell_CD56+107a+_	6B11+CD56+CD107a+	NKcell	iNKTcell_CD56+107a+_/NKcell	[20]

**Table 2:** Characteristics of acute myeloid leukemia (AML) patients and healthy volunteers are presented.

FAB/WHO Classification *	Stage	Patient	Age at Diagn.	Sex	ELN Risk Stratification	Blasts Phenotype (CD)	Blast in PB (%) **	WBC in PB (/nl) *	PLT in PB (/nl) *	Hemoglobin in PB (g/dl) *	Conducted Cell Biological Experiments	Sources for VOC Analyses (Supernatant-Derived VOCs)
pAML	First diagnosis	P1567	98	f	very unfavourable	**34**,**117**,15,65	16	7.96	12	8.3	DC, MLC, CTX, Deg, InCyt	Serum, DCD0 M/0, DCDE M, DCDE 0
pAML/M5		P1572	63	f	very unfavourable	**34**,**117**,65,33,13	12	1.87	77	9.4	DC, MLC, CTX, Deg, InCyt	Serum, DCD0 M/0, DCDE M/0, MLCD0 M/0, MLCDE M/0
pAML/M6		P1573	61	m	unfavourable	**34**,**117**,65,13,71	13	3.8	19	7.7	DC, MLC, CTX, Deg, InCyt	Serum, DCD0 M/0, DCDE M/0, MLCD0 M/0, MLCDE M/0
pAML/M4		P1581	56	m	unfavourable	**34**,**117**,33,13,15	58	31.4	21	8.3	DC, MLC, CTX, Deg, InCyt	Serum, DCD0 M/0, DCDE M/0, MLCD0 M/0, MLCDE M/0
pAML/M3		P1602	67	m	favourable	**117**,**34**,33,13,56	16	3.4	162	9.1	DC, MLC, CTX	Serum, DCD0 M/0, DCDE M/0, MLCD0 M/0, MLCDE M/0
sAML		P1604	60	f	intermediate	**117**,**34**,13,33,65,7	16	0.21	16	6	DC, MLC, CTX, Deg, InCyt	Serum, DCD0 M/0, DCDE M/0, MLCD0 M/0, MLCDE M/0
pAML/M4		P1630	29	m	favourable	**34**,**117**,13,33,15,64	16	24.56	117	14.5	DC, MLC, CTX, Deg, InCyt	Serum, DCD0 M/0, DCDE M/0, MLCD0 M/0, MLCDE M/0
pAML/M2		P1635	51	m	intermediate	**34**,117,**15**,33	25	0.6	53	7.6	DC, MLC, Deg, InCyt	Serum, DCD0 M/0
pAML		P1638	68	m	unfavourable	**34**,**117**,33,13,56,4,71	60	9.8	20	7.9	DC, MLC, CTX, Deg, InCyt	Serum, DCD0 M/0, DCDE M/0, MLCD0 M/0, MLCDE M/0
pAML/M4	Persisting	P1594	70	f	favourable	**34**,**117**,65,33,13	11	1.32	232	11.9	DC, MLC, CTX, Deg, InCyt	Serum, DCD0 M/0, DCDE M/0, MLCD0 M/0
pAML	disease	P1595	50	f	intermediate	**34**,**117**,65,13,33,56	15	3	43	7.4	DC, MLC, CTX	Serum, DCD0 M/0, DCDE M/0, MLCD0 M/0, MLCDE M/0
sAML		P1597	83	f	intermediate	**117**,56,**34**,15,65,33	54	88.6	41	11.3	DC, MLC, CTX, Deg, InCyt	Serum, DCD0 M, DCDE M/0
pAML		P1603	32	f	unfavourable	**34**,**117**,15,33	50	0.71	25	6	DC, MLC, CTX, Deg, InCyt	Serum, DCD0 M/0, DCDE M/0, MLCD0 M/0, MLCDE M/0
pAML		P1616	69	f	intermediate	**34**,**117**,33	16	1.01	86	9.1	-	Serum, DCD0 M/0, DCDE M/0, MLCD0 M/0, MLCDE M/0
sAML	Relapse after	P1598	61	f		**117**,**34**,33,13	25	19.6	15	8.3	DC, MLC, CTX, Deg, InCyt	Serum, MLCD0 M/0, MLCDE M/0
pAML/M4	stem cell	P1599	71	f		**34**,**117**,33,7,13	79	54.8	180	9.2	DC, MLC, CTX, Deg	Serum, DCD0 M/0, DCDE M/0, MLCD0 M/0, MLCDE M/0
pAML	transplantation	P1632	56	f		**34**,13,65,33,56,**117**	65	21.73	40	14.1	DC, MLC, CTX, Deg, InCyt	Serum, DCD0 M/0, DCDE M/0, MLCD0 M/0, MLCDE M/0
Healthy		P1566	54	f							DC, MLC, Deg, InCyt	Serum, DCD0 M/0, DCDE M/0, MLCD0 M/0, MLCDE M/0
		P1579	30	m							DC, MLC, Deg, InCyt	Serum, DCD0 M/0, DCDE M/0, MLCD0 M/0, MLCDE M/0
		P1580	24	f							DC, MLC, Deg, InCyt	Serum, DCD0 M/0, DCDE M/0, MLCD0 M/0, MLCDE M/0
		P1582	27	m							DC, MLC, Deg, InCyt	Serum, DCD0 M/0, DCDE M/0, MLCD0 M/0, MLCDE M/0
		P1583	28	m							DC, MLC, Deg, InCyt	Serum, DCD0 M/0, DCDE M/0, MLCD0 M/0, MLCDE M/0
		P1585	29	m							DC, MLC, Deg, InCyt	Serum, DCD0 M/0, DCDE M/0, MLCD0 M/0, MLCDE M/0
		P1586	29	m							DC, MLC	Serum, DCDE M/0, MLCD0 M/0, MLCDE M/0
		P1590	23	f							DC, MLC	Serum, DCD0 M/0, DCDE M/0, MLCD0 M/0, MLCDE M/0
		P1592	58	f							DC, MLC, Deg, InCyt	Serum, DCD0 M/0, DCDE M/0, MLCD0 M/0, MLCDE M/0
		P1596	26	m							DC, MLC	Serum, DCD0 M/0, DCDE M/0, MLCD0 M/0, MLCDE M/0
		P1611	27	f							DC, MLC, Deg, InCyt	Serum, DCD0 M/0, DCDE M/0, MLCD0 M/0, MLCDE M/0
		P1613	24	f							DC, MLC, Deg, InCyt	Serum, DCD0 M/0, DCDE M/0, MLCD0 M/0, MLCDE M/0
		P1636	22	m							DC, MLC, Deg, InCyt	Serum, DCD0 M/0, DCDE M/0, MLCD0 M/0, MLCDE M/0
		P1637	22	f							DC, MLC, Deg, InCyt	Serum, DCD0 M/0, DCDE M/0, MLCD0 M/0, MLCDE M/0

FAB classification, French American British classification of acute myeloid leukemia; WHO classification, World Health Organization classification of AML; ELN, EuropeanLeukemiaNet; VOC, volatile organic compounds; WBC, white blood cells; PLT, platelets/thrombocytes; f, female; m, male; DC, dendritic cell culture measurements; MLC, mixed lymphocyte culture measurement; CTX, cytotoxicity measurements; Deg, degranulation assay; InCyt, intracellular cytokine assay; bold text indicates the blast markers used for DC_leu_ evaluation; * indicates the FAB types of patients, given where available. If they were not available (or not performed) only ‘pAML/sAML’ is given; ** indicates a clinical parameter on the day of sampling.

## Data Availability

The data presented in this study are available in this article.
